# Multiscale Computational Prioritization of Chromone-Based Acetylcholinesterase Ligands

**DOI:** 10.3390/ijms27209153

**Published:** 2026-10-15

**Authors:** Oussama Khibech, Bahia Abdelfattah, Mohamed Bouhrim, Moneerah J. Alqahtani, Jawaher H. Alqahtani, Joe Miantezila Basilua, Aziz Boulouiz, Rachid Touzani

**Affiliations:** 1 Laboratory of Materials Chemistry and Applications (LCMA), Department of Chemistry, Faculty of Sciences, Mohammed Premier University, BP 717, Oujda 60000, Morocco; oussama.khibech.d24@ump.ac.ma (O.K.);; 2 Laboratory of Physical Chemistry of Materials, Natural Substances and Environment, Chemistry Department, Faculty of Sciences and Techniques of Tangier, Tangier 90090, Morocco; 3 Biological Engineering Laboratory, Faculty of Sciences and Techniques, Sultan Moulay Slimane University, Mghila, P.O. Box 523, Beni Mellal 23000, Morocco; m.bouhrim@usms.ma; 4 Department of Pharmacognosy, College of Pharmacy, King Saud University, P.O. Box 2457, Riyadh 11451, Saudi Arabia; 5 BioSTM—UR 7537 Biostatistics, Processing and Modelling of Biological Data, Université Paris Cité, 75006 Paris, France

**Keywords:** acetylcholinesterase, chromone derivatives, molecular docking, molecular dynamics, MM/PBSA, SDG3

## Abstract

Identifying acetylcholinesterase (AChE) ligands for Alzheimer’s disease research requires integrating molecular recognition with pharmacokinetic and safety considerations. Here, four designed chromone derivatives (M1–M4) were investigated to determine how conformational sampling and complementary molecular descriptors refine docking-based prioritization. Donepezil redocking in human AChE and cross-docking into *Torpedo californica* AChE recovered the crystallographic and transferred reference orientations, respectively, with RMSDs of 0.942 and 1.074 Å. Molecular docking was integrated with 800 ns molecular dynamics, MM/PBSA, electronic-structure calculations, and pharmacokinetic/toxicity predictions. M4 achieved the most favorable chromone docking score (−9.6 kcal mol^−1^), whereas M3 yielded the most favorable chromone MM/PBSA estimate within the 775–800 ns window (−23.90 ± 2.31 kcal mol^−1^). Donepezil retained a more favorable MM/PBSA estimate (−41.00 ± 2.75 kcal mol^−1^). M4 exhibited the highest electronic hardness, while predicted toxicity profiles revealed distinct liabilities across the series. The divergence between docking and sampled energetics highlights the value of integrating complementary criteria when selecting compounds for experimental evaluation. These protocol-dependent findings prioritize M3 and M4 as complementary computational candidates requiring experimental confirmation of AChE inhibition, selectivity, and safety.

## 1. Introduction

Alzheimer’s disease (AD) is the world’s leading cause of dementia, and demographic studies show its prevalence is accelerating as populations age. Recent estimates indicate that 6.9 million Americans aged ≥ 65 years were living with Alzheimer’s dementia in 2024, with projections of 13.8 million by 2060; in parallel, dementia burden is expected to continue rising globally with population aging [1,2,3]. Early cognitive decline is driven by a marked loss of cholinergic neurotransmission in the hippocampus and neocortex, a discovery that inspired the “cholinergic hypothesis” and firmly established acetylcholinesterase (AChE) as a proven therapeutic target [4]. Current AChE inhibitors (donepezil, rivastigmine and galantamine) elevate synaptic acetylcholine and slow symptom progression, but their modest efficacy, narrow therapeutic window, and gastrointestinal and cardiovascular side effects emphasize the need for safer, longer-acting drugs [5,6]. Mixed neuropathologies in the aging brain further complicate therapeutic targeting [7].

**Figure 1:**
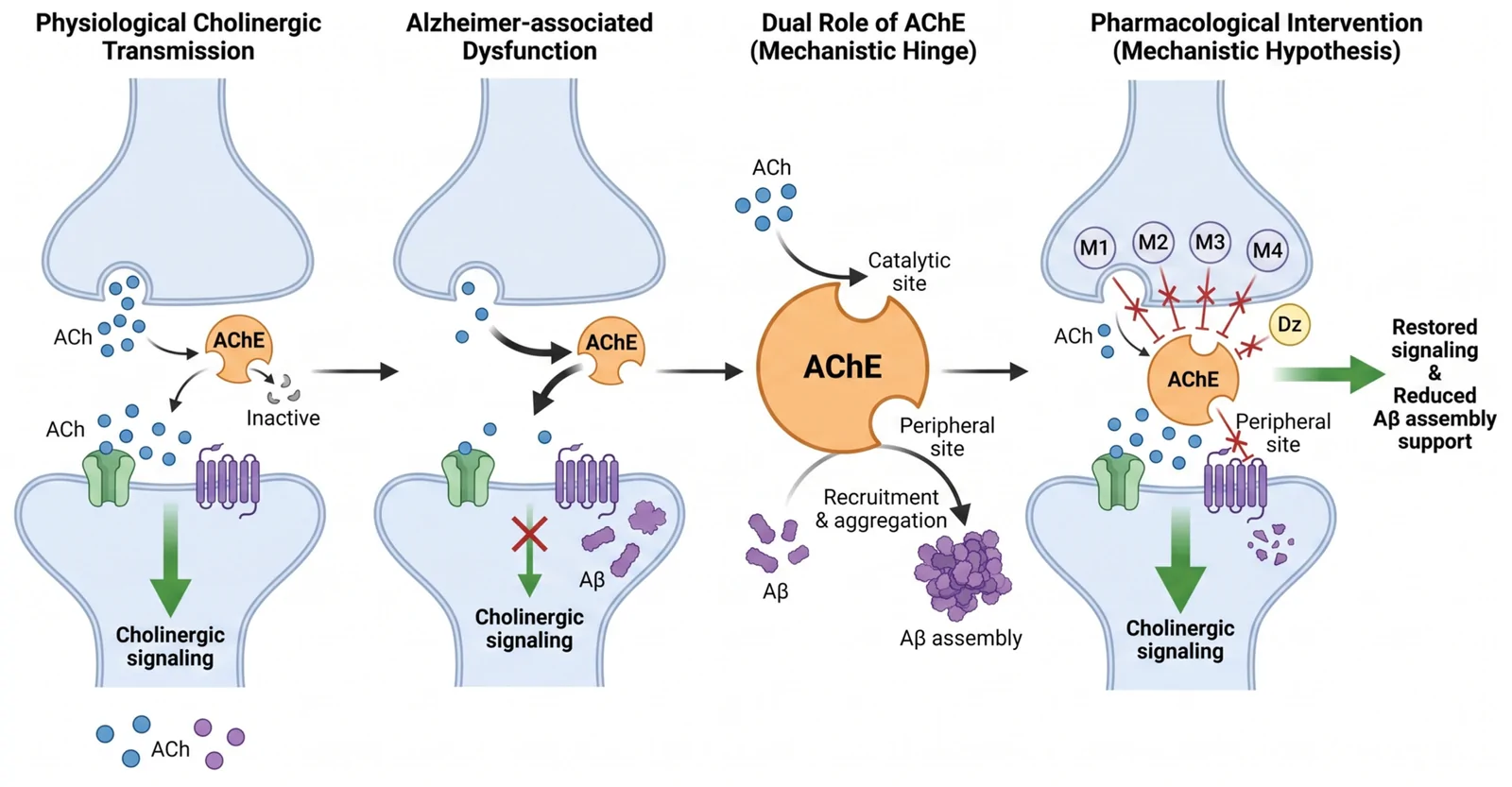
Cellular pathophysiology of cholinergic failure and the pharmacological rationale motivating the evaluation of M1–M4 as potential acetylcholinesterase ligands, with donepezil (Dz) the as reference.

Creating next-generation AChE inhibitors therefore demands a delicate balance of high binding affinity, efficient blood–brain barrier penetration, metabolic stability, and low off-target toxicity [8]. Polyphenolic frameworks, especially flavonoids, offer a compelling starting point, combining antioxidant, anti-inflammatory, and metal-chelating activities with multi-pathway neuroprotection [9,10]. Native flavonoids, however, suffer from poor water solubility, rapid phase-II metabolism, and insufficient potency within the AChE catalytic gorge. Medicinal-chemistry efforts thus focus on rational derivatization: halogenation to boost lipophilicity, O-methylation to protect phenolic hydroxyls from glucuronidation, and the addition of bulky aromatic groups to reinforce π-π stacking along the enzyme’s aromatic channel [11,12,13]. Structure–activity relationship (SAR) work consistently demonstrates that extended conjugated systems improve binding, while carefully positioned polar substituents can form stabilizing hydrogen bonds in the peripheral anionic site, anchoring inhibitors and limiting amyloid-β aggregation [14]. The disease-level rationale for this dual-site inhibition strategy is summarized in Figure 1.

Computer-aided drug-design (CADD) workflows combine docking and molecular dynamics to examine protein–ligand recognition [15]. Web-based tools such as SwissADME and pkCSM rapidly predict physicochemical descriptors, rule-of-five compliance, intestinal absorption, blood–brain barrier permeability, cytochrome-P450 liabilities and renal clearance, while ProTox-III extends this in silico triage to acute oral toxicity and mechanistic safety alerts [16,17,18]. By weeding out weak candidates before synthesis, these filters shorten development timelines and cut costs. In parallel, state-of-the-art docking engines such as AutoDock Vina can rank vast libraries against high-resolution AChE structures in hours, and explicit-solvent molecular-dynamics (MD) simulations now routinely probe complex stability and induced fit over hundreds of nanoseconds [19,20].

**Figure 2:**
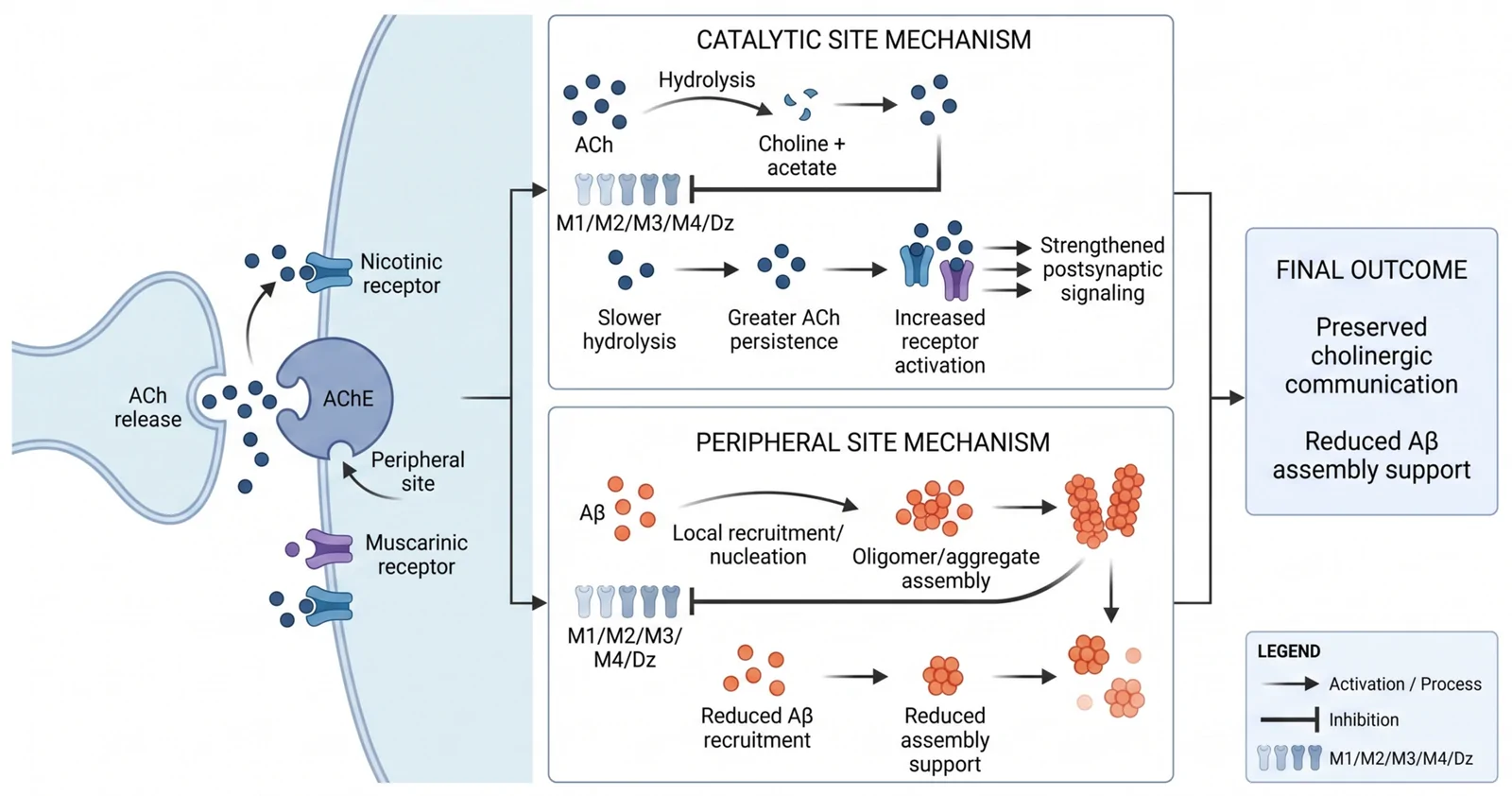
Proposed functional consequences motivating catalytic-site and peripheral-site acetylcholinesterase targeting: preservation of synaptic acetylcholine and attenuation of Aβ assembly support. M1–M4 are hypothetical candidates, whereas donepezil (Dz) is the pharmacological reference.

Recent reviews on dual AChE/MAO-B multitarget ligands highlight benzopyranone scaffolds (including chromones/coumarins) as privileged cores. Across these series, a planar aromatic axis supports π-stacking along the AChE gorge, while strategically positioned acceptors and compact hydrophobic substituents tune catalytic-site anchoring and peripheral-site recognition [21,22]. Accordingly, the design of M1–M4 followed a structure-guided medicinal-chemistry strategy aimed at exploring possible catalytic-site (CAS) and peripheral-site (PAS) recognition. The chromone nucleus was selected as a rigid, π-conjugated scaffold intended to support aromatic interactions within the gorge while maintaining a molecular size compatible with blood–brain barrier permeation. Guided by these principles, M1–M4 chromone derivatives were constructed as a focused neutral set (single microspecies at pH 7.4), in which substituent variation was deliberately performed to modulate three key parameters: hydrogen-bond acceptor density for oxyanion-hole engagement, hydrophobic surface extension to reinforce π-π stacking along the aromatic channel, and electronic distribution to optimize electrophilicity and frontier-orbital alignment. Stepwise substitution was intentionally introduced to explore structure-dependent recognition hypotheses; cooperative CAS–PAS inhibition was not experimentally established. The functional linkage between catalytic-site inhibition, peripheral-site blockade, synaptic acetylcholine preservation, and reduced Aβ assembly support is illustrated in Figure 2.

**Figure 3:**
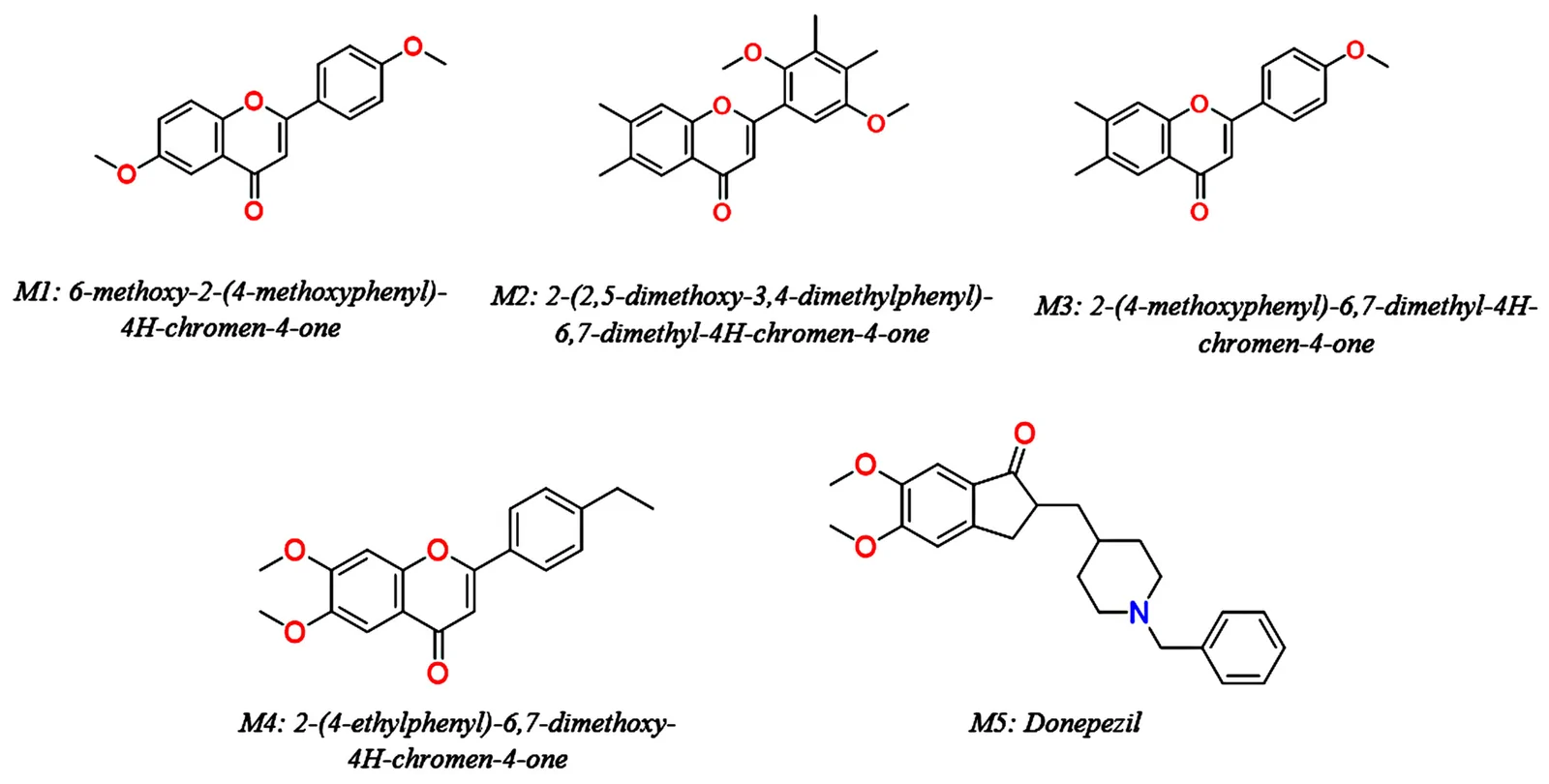
Chemical structures of chromone derivatives M1–M4 and the reference drug donepezil.

Here, we designed and virtually evaluated chromone-based scaffolds (Figure 3) for predicted AChE gorge accommodation, with donepezil (Dz) as a pharmacological comparator and an experimental pose reference for docking validation. The workflow integrated ADME/toxicity screening, structure-guided pose benchmarking, docking, 800 ns molecular dynamics, and terminal-window MM/PBSA, PCA/FEL, DCCM, and DFT. The objective was to identify compounds that combine persistent gorge accommodation with a defensible energetic, dynamical, electronic, and safety profile, while explicitly distinguishing docking rank from solution-phase ensemble behavior and treating all predictions as hypotheses for experimental validation.

## 2. Results

### 2.1. Docking Validation

**Figure 4:**
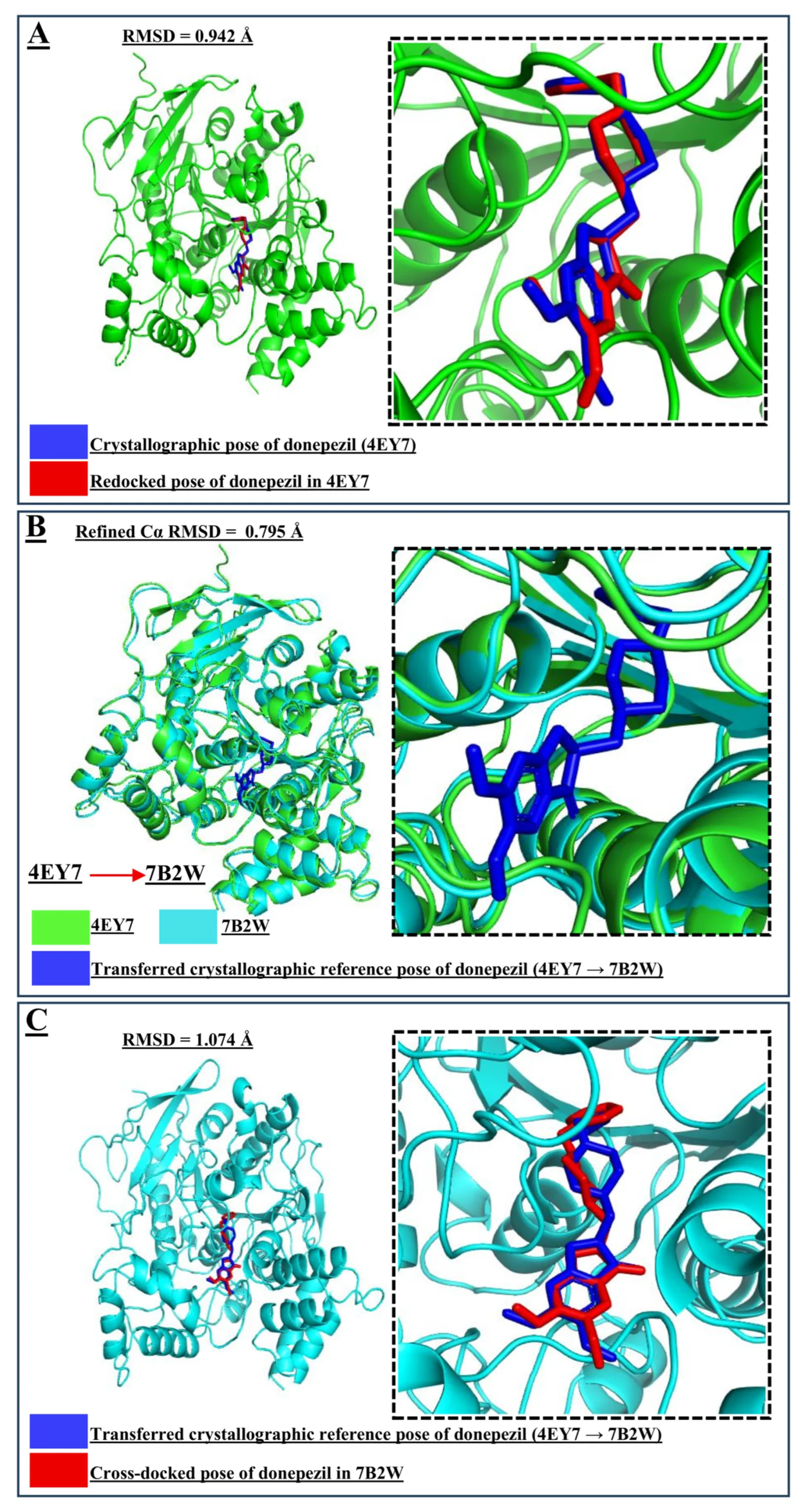
Donepezil redocking and cross-docking validation. (**A**) Crystallographic (blue) and redocked (red) donepezil in human AChE 4EY7 (green): heavy-atom RMSD, 0.942 Å. (**B**) Alignment of 4EY7 (green) to *Torpedo californica* AChE 7B2W (cyan): refined Cα RMSD, 0.795 Å; crystallographic donepezil (blue) was transferred into the 7B2W coordinate frame. (**C**) Transferred reference (blue) and cross-docked donepezil (red) in 7B2W: heavy-atom pose RMSD, 1.074 Å. The transferred reference is not an experimentally determined donepezil pose in 7B2W.

Donepezil redocking in human AChE (PDB 4EY7) reproduced the crystallographic pose with a heavy-atom RMSD of 0.942 Å. Structural alignment of 4EY7 to *Torpedo californica* AChE 7B2W gave a refined Cα RMSD of 0.795 Å, and cross-docking of donepezil into 7B2W reproduced the transferred reference orientation with a heavy-atom RMSD of 1.074 Å (Figure 4).

### 2.2. ADME Results

#### 2.2.1. In Silico Drug-Likeness, Brain Permeability and ADME Profile

SwissADME predicted molecular weights of 282.29, 338.40, 280.32, and 310.34 g mol^−1^ for M1–M4, respectively, compared with 379.49 g mol^−1^ for donepezil. M1–M4 had 2–4 rotatable bonds, TPSA values of 39.44–48.67 Å^2^, cLogP values of 3.15–4.36, no Lipinski or Veber violations, and a predicted bioavailability score of 0.55 (Table 1).

**Figure 5:**
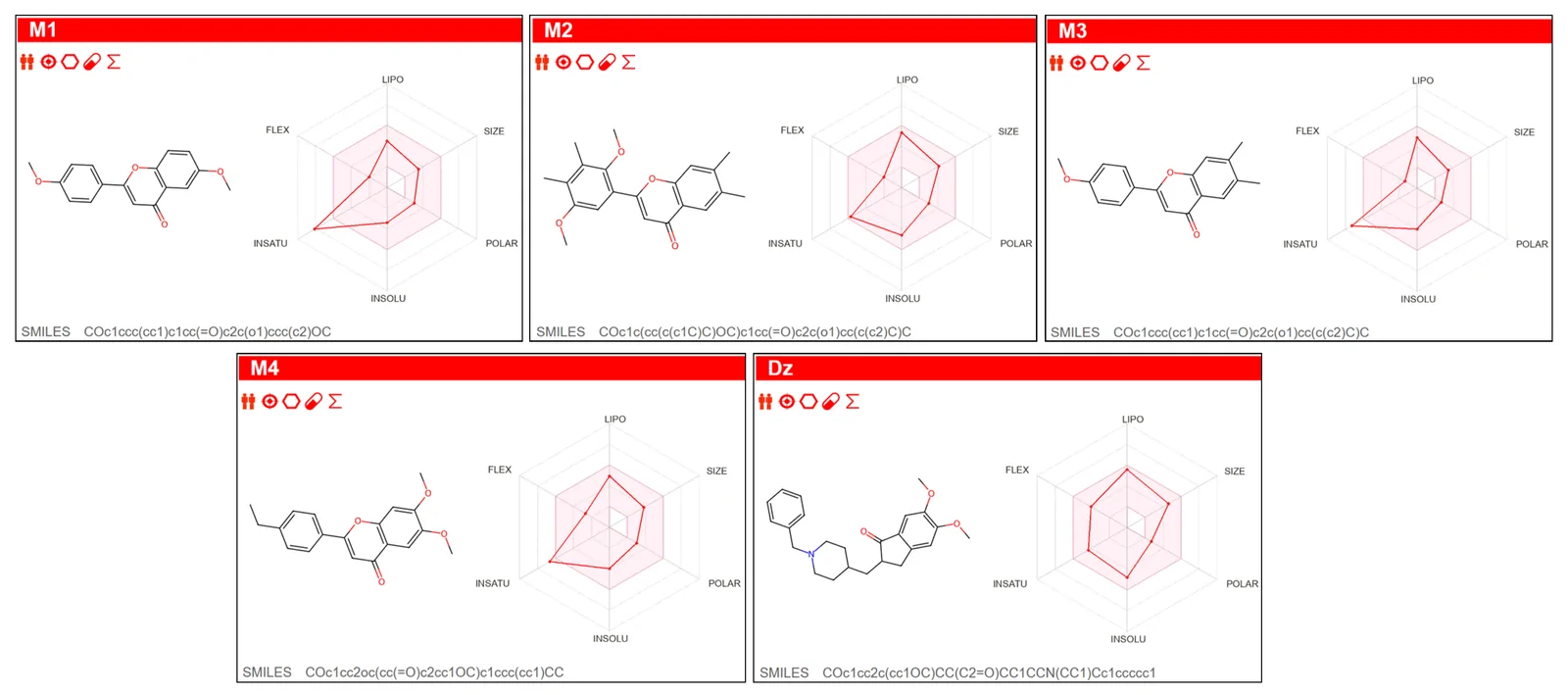
Chemical structures, SMILES representations, and SwissADME bioavailability-radar profiles of chromone derivatives M1–M4 and the reference inhibitor donepezil (Dz). The structures and SMILES describe the parent representations submitted for ADME prediction.

The chemical structures, SMILES representations, and SwissADME bioavailability-radar profiles of M1–M4 and donepezil are presented in Figure 5.

**Figure 6:**
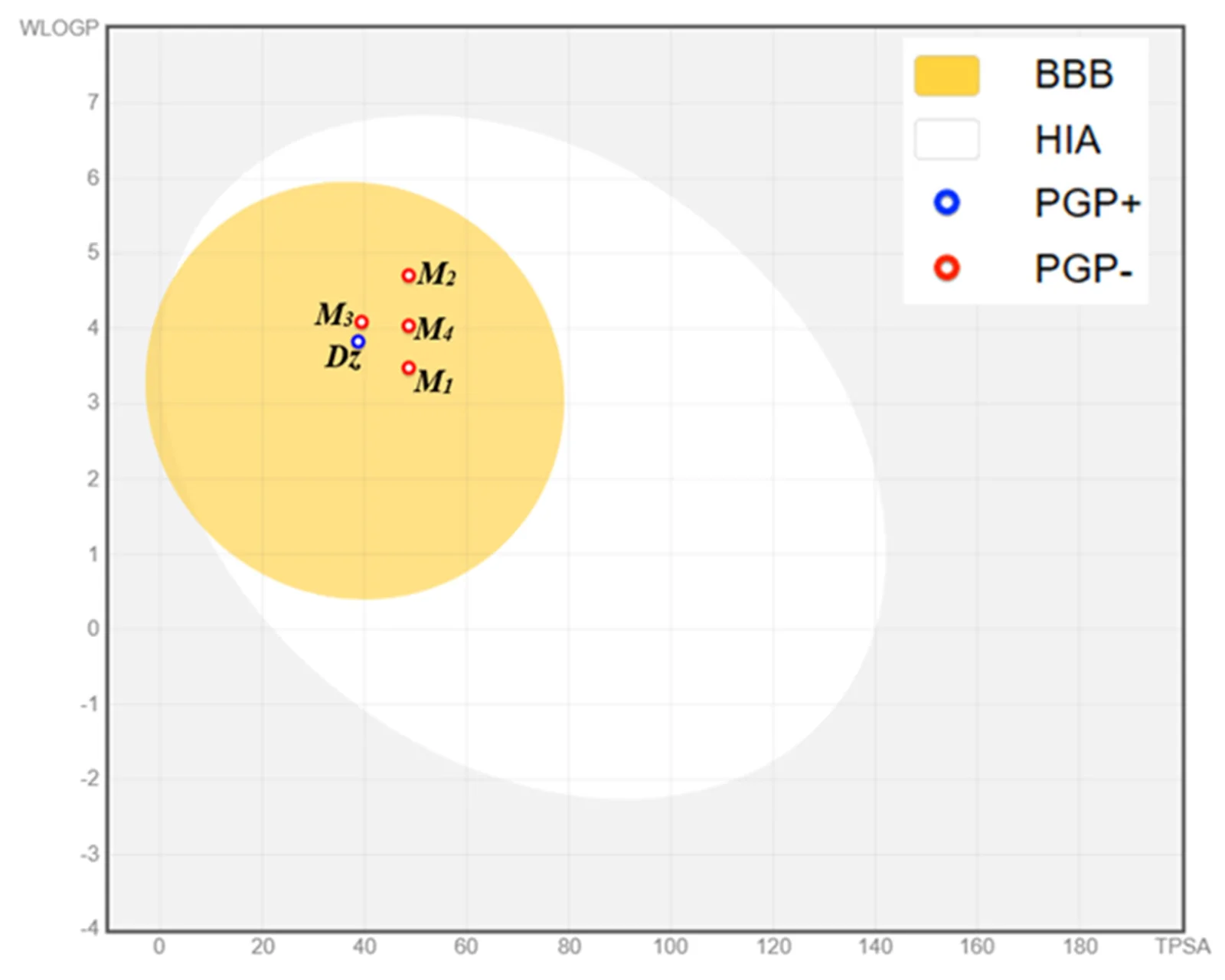
SwissADME BOILED-Egg plot illustrating gastrointestinal absorption, BBB permeability and P-gp substrate classification for chromone derivatives M1–M4 versus the results for the reference inhibitor donepezil.

In the BOILED-Egg model, M1–M4 were located in the regions associated with high gastrointestinal absorption and passive blood–brain barrier permeation. Donepezil was predicted as a P-glycoprotein substrate, whereas M1–M4 were predicted as non-substrates (Figure 6).

#### 2.2.2. pkCSM Predictive Pharmacokinetic Analysis: Absorption, Distribution and Brain Penetration

**Figure 7:**
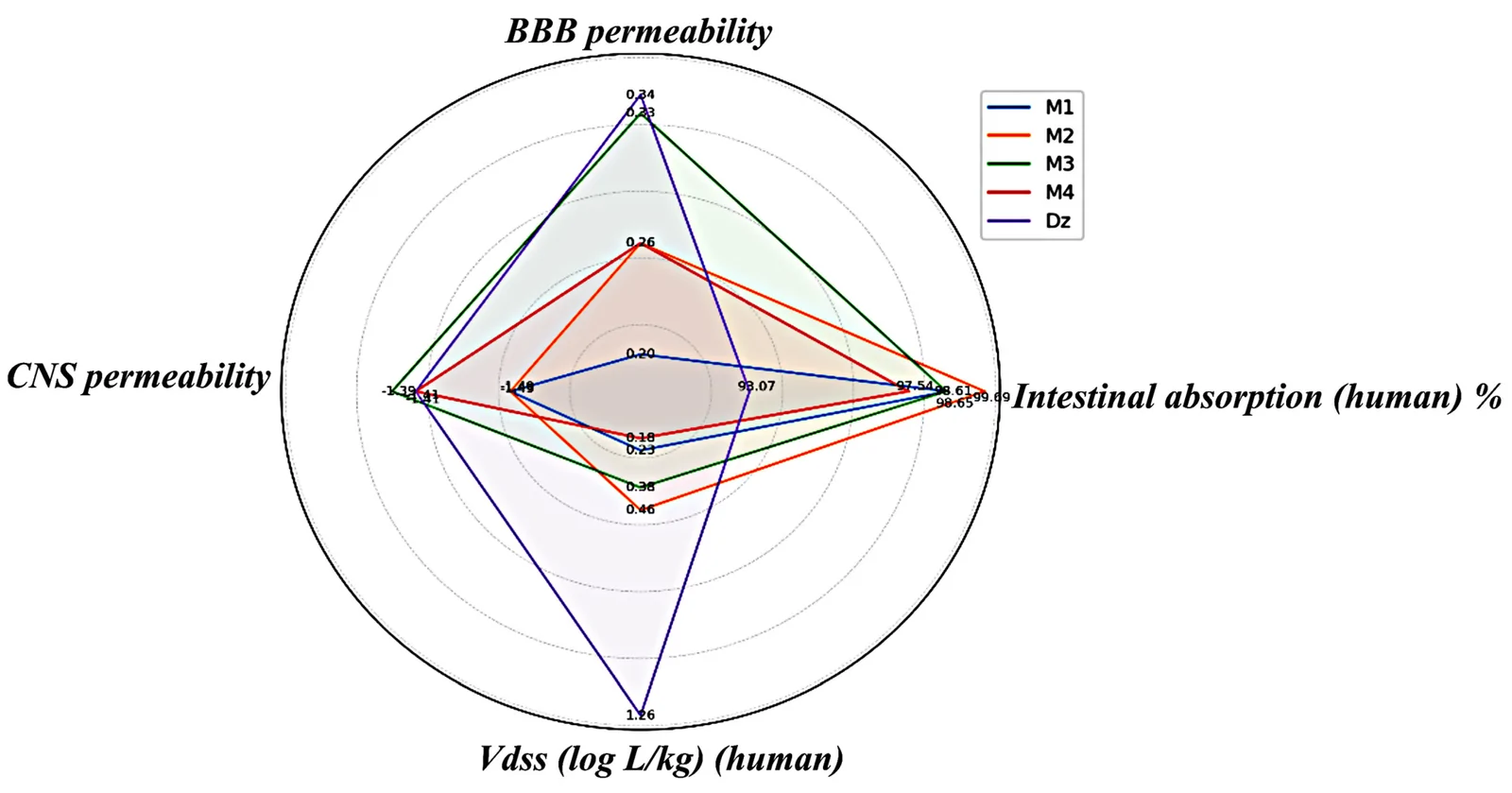
Absolute-scale radar plot of key pkCSM-predicted ADME parameters for chromone derivatives (M1–M4) compared with that for the reference inhibitor donepezil (Dz).

pkCSM predicted intestinal absorption above 97% for M1–M4 compared with approximately 93% for donepezil. Predicted LogBB values were 0.20 for M1, 0.26 for M2, 0.33 for M3, 0.26 for M4, and 0.34 for donepezil. logPS values were approximately −1.4 across the chromone series, while logVdss ranged from 0.18 to 0.46, with M2 showing the highest value (0.463) and M4 the lowest (0.183) (Figure 7).

#### 2.2.3. pkCSM-Based Metabolic and Clearance Predictions

M1–M4 were predicted to be neither CYP2D6 substrates nor inhibitors, whereas donepezil was predicted to be a CYP2D6 inhibitor. All five compounds were predicted as CYP3A4 substrates, while only donepezil was classified as a CYP3A4 inhibitor. All compounds were predicted as renal OCT2 substrates. Predicted total-clearance values were 0.676, 0.657, 0.614, and 0.447 log mL min^−1^ kg^−1^ for M1–M4, respectively, compared with 0.998 for donepezil (Table 2).

### 2.3. ProTox-III Acute and Mechanistic Toxicity Predictions

**Figure 8:**
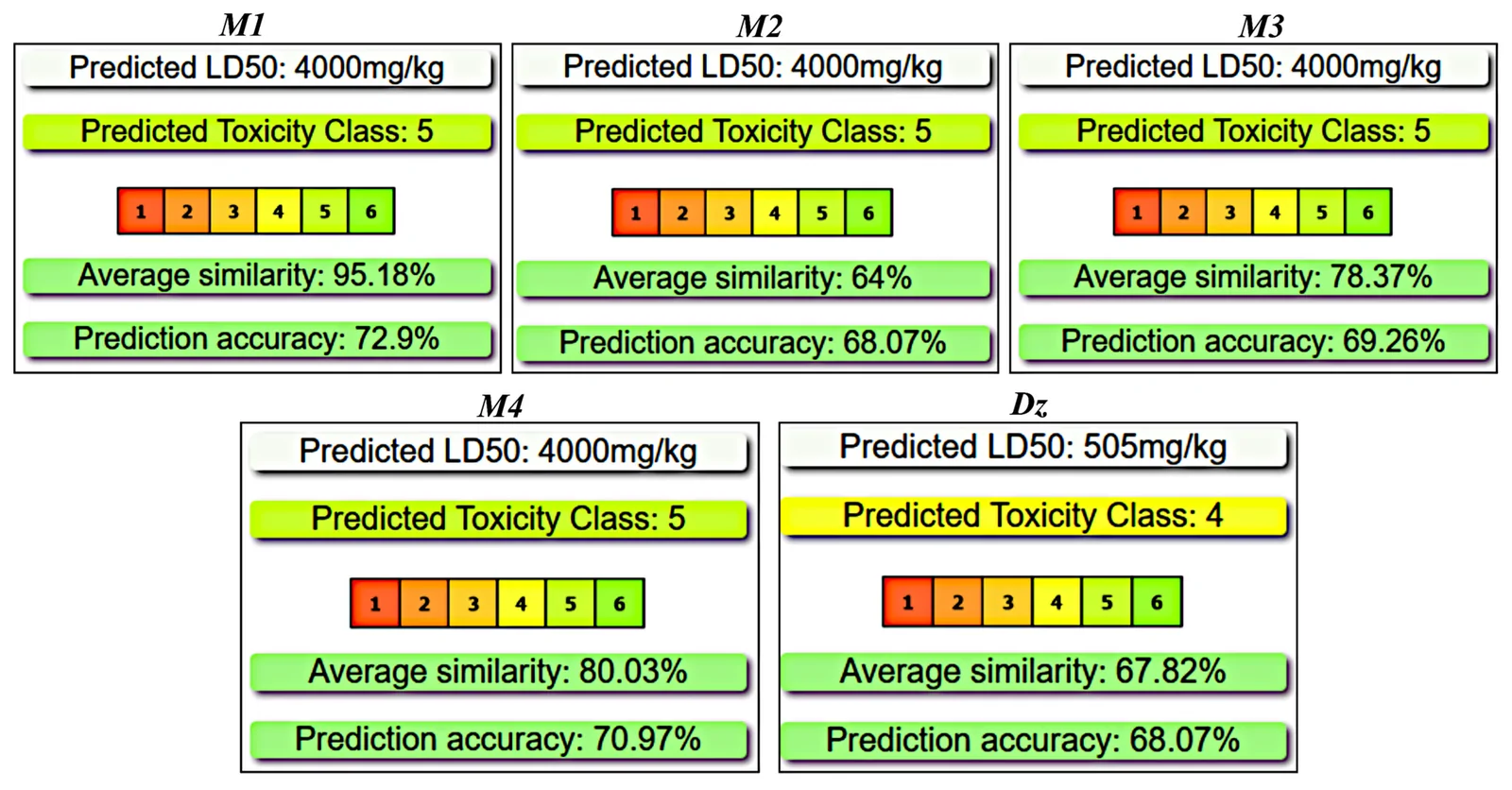
ProTox-III-predicted acute oral toxicity for chromone analogues (M1–M4) versus that for donepezil (Dz): LD_50_, GHS toxicity class, structural similarity and model accuracy.

ProTox-III assigned M1–M4 a predicted oral LD_50_ of 4000 mg kg^−1^ and a GHS toxicity of class 5, whereas donepezil was assigned 505 mg kg^−1^ and class 4. Average structural similarity/model accuracy values were 95.18%/72.90% for M1, 64.00%/68.07% for M2, 78.37%/69.26% for M3, and 80.03%/70.97% for M4 (Figure 8).

Target-level predictions showed distinct compound-specific patterns across the results for organ toxicity, toxicity endpoints, Tox21 nuclear-receptor and stress-response pathways, molecular initiating events, and CYP-related metabolism. The complete active/inactive classifications and associated probabilities are reported in Table 3.

### 2.4. Molecular Docking

AutoDock Vina scores were −9.0 kcal mol^−1^ for M1, −9.5 for M2, −9.2 for M3, −9.6 for M4, and −9.3 for donepezil. Reported polar contacts were Tyr130/Glu199/Ser200 for M1, Gly117/Gly118/Tyr121/Glu199 for M4, and Tyr121/Arg289 for donepezil (Table 4).

**Figure 9:**
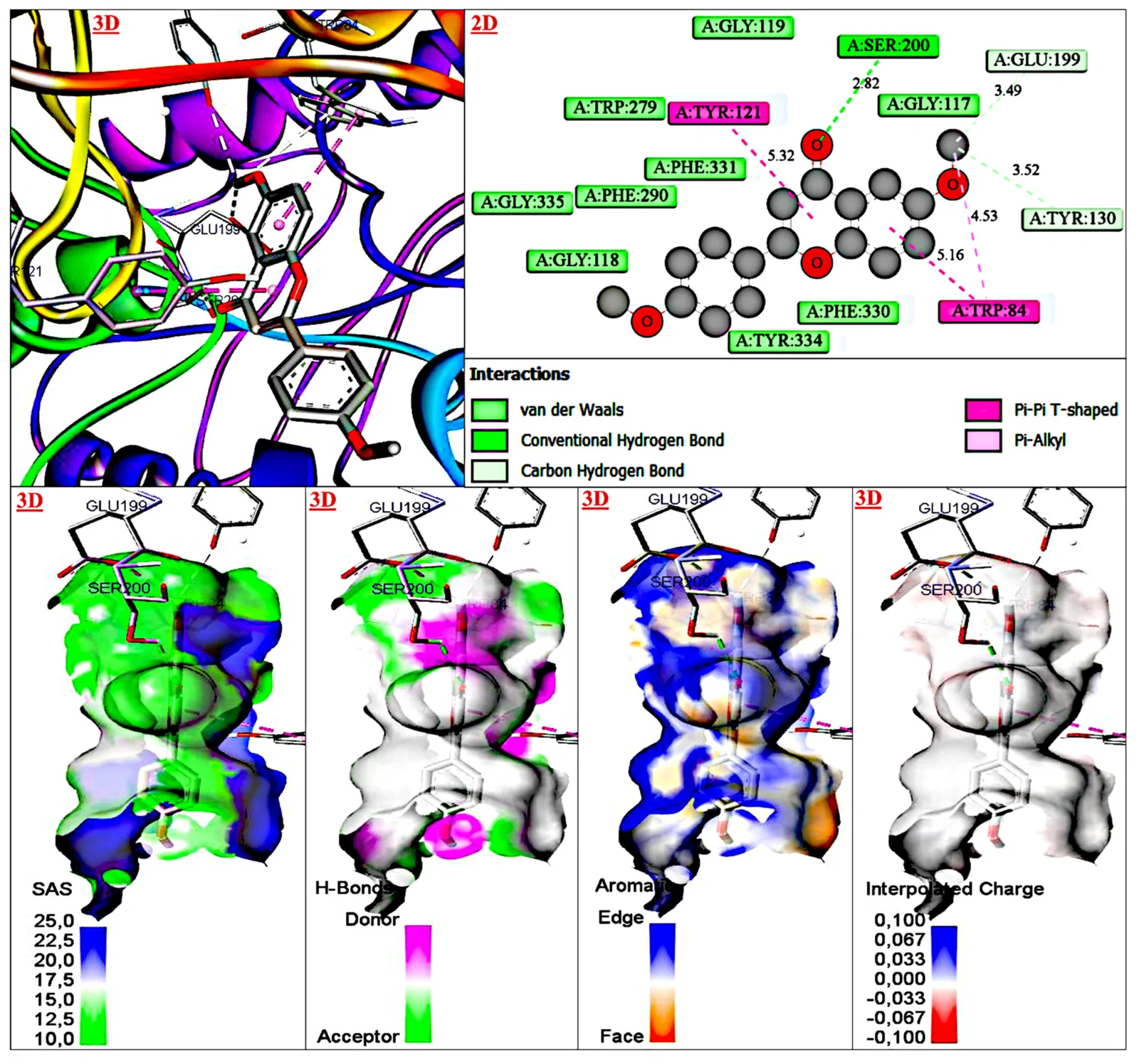
Three-dimensional/two-dimensional interaction diagrams and surface property maps (SAS, H-bond donor/acceptor, aromatic edge/face, interpolated charge) for ligand M1 bound to the 7B2W active site.

**Figure 10:**
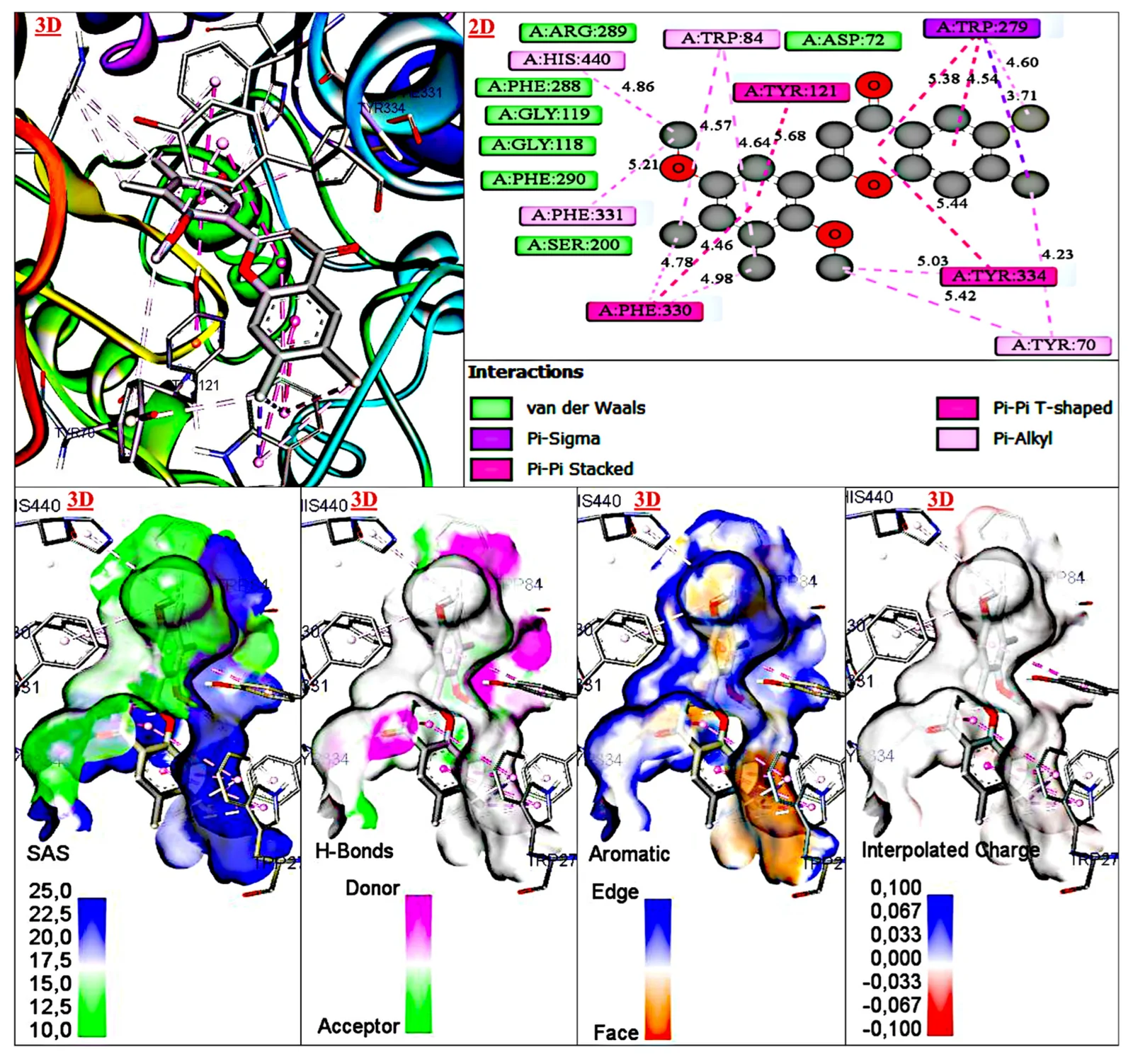
Three-dimensional/two-dimensional interaction diagrams and surface property maps for ligand M2 bound to the 7B2W active site.

**Figure 11:**
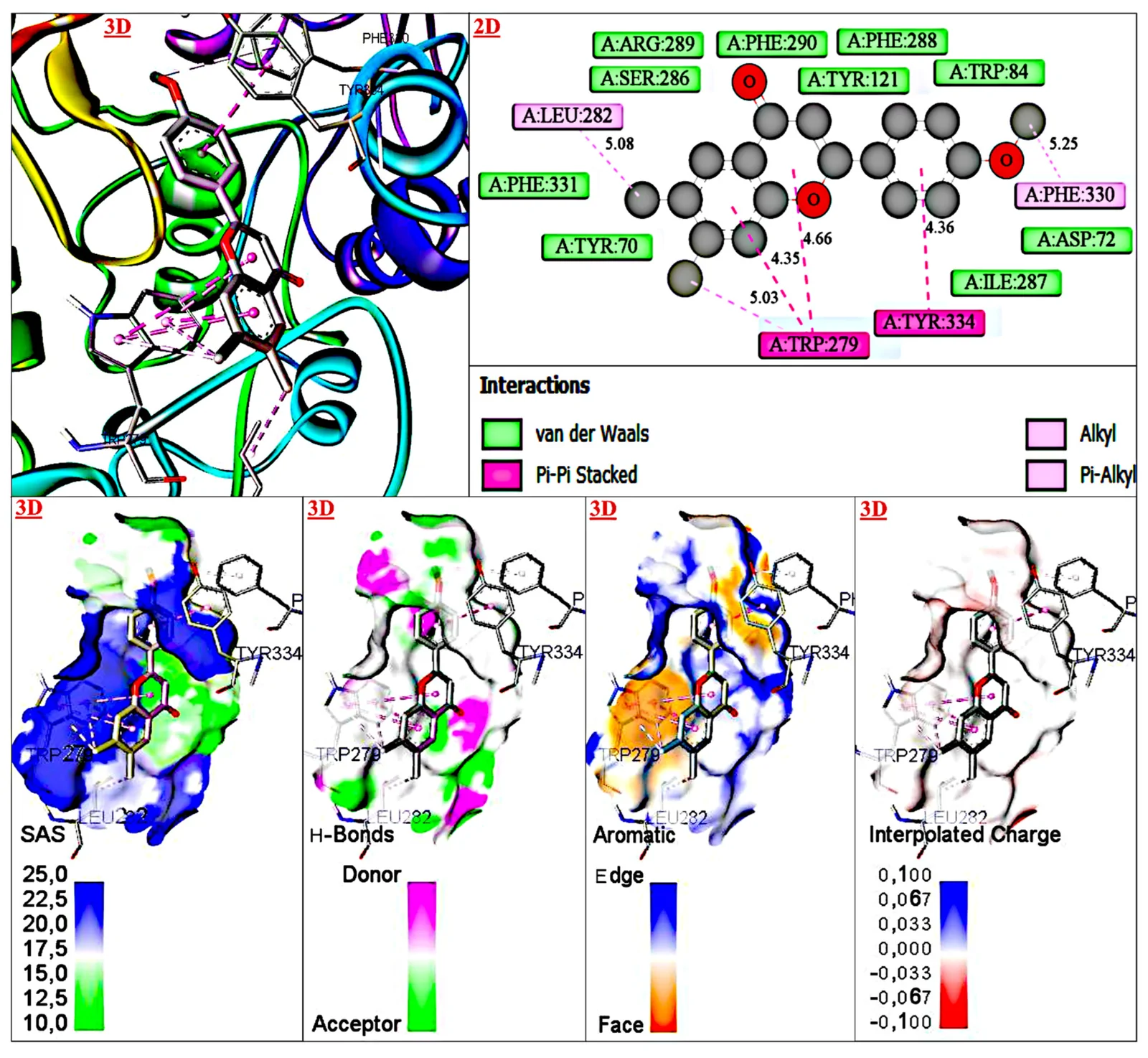
Three-dimensional/two-dimensional interaction diagrams and surface property maps for ligand M3 bound to the 7B2W active site.

**Figure 12:**
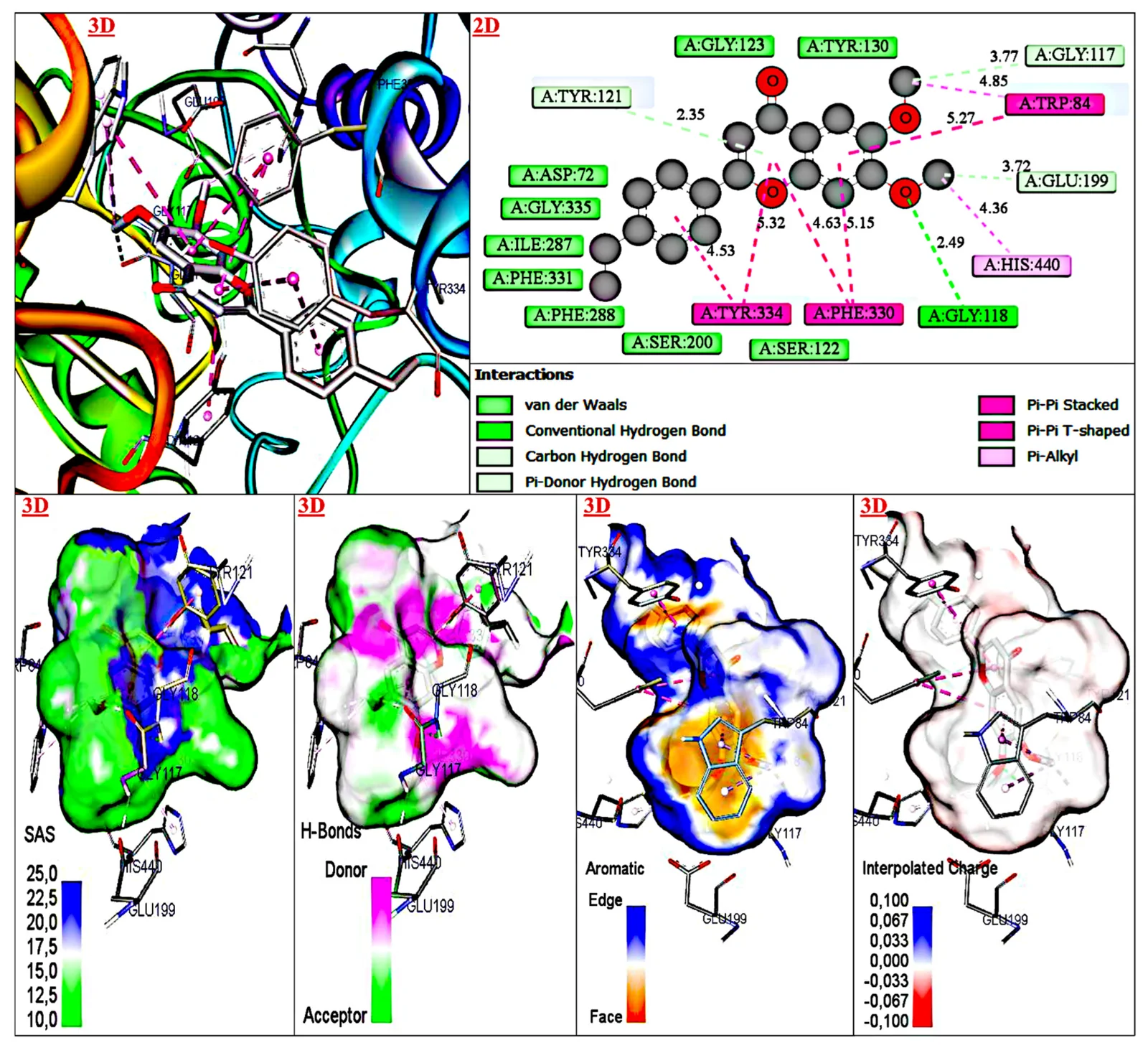
Comprehensive 2-D/3-D interaction profile of chromone derivative M4 bound to acetylcholinesterase: hydrogen-bond network, π-stacking, van der Waals shell, and surface complementarity.

Interaction maps showed aromatic and hydrophobic contacts throughout the AChE gorge for M1–M4. M1 displayed π interactions with Trp84 and Tyr131; M2 showed a denser aromatic interaction network involving Trp279, Tyr121, Phe330, and Tyr334; M3 showed π-stacking with Trp279 and Tyr334; and M4 displayed the richest reported polar-contact pattern, together with aromatic and hydrophobic interactions (Figure 9, Figure 10, Figure 11 and Figure 12).

### 2.5. Long-Timescale Molecular Dynamics

**Figure 13:**
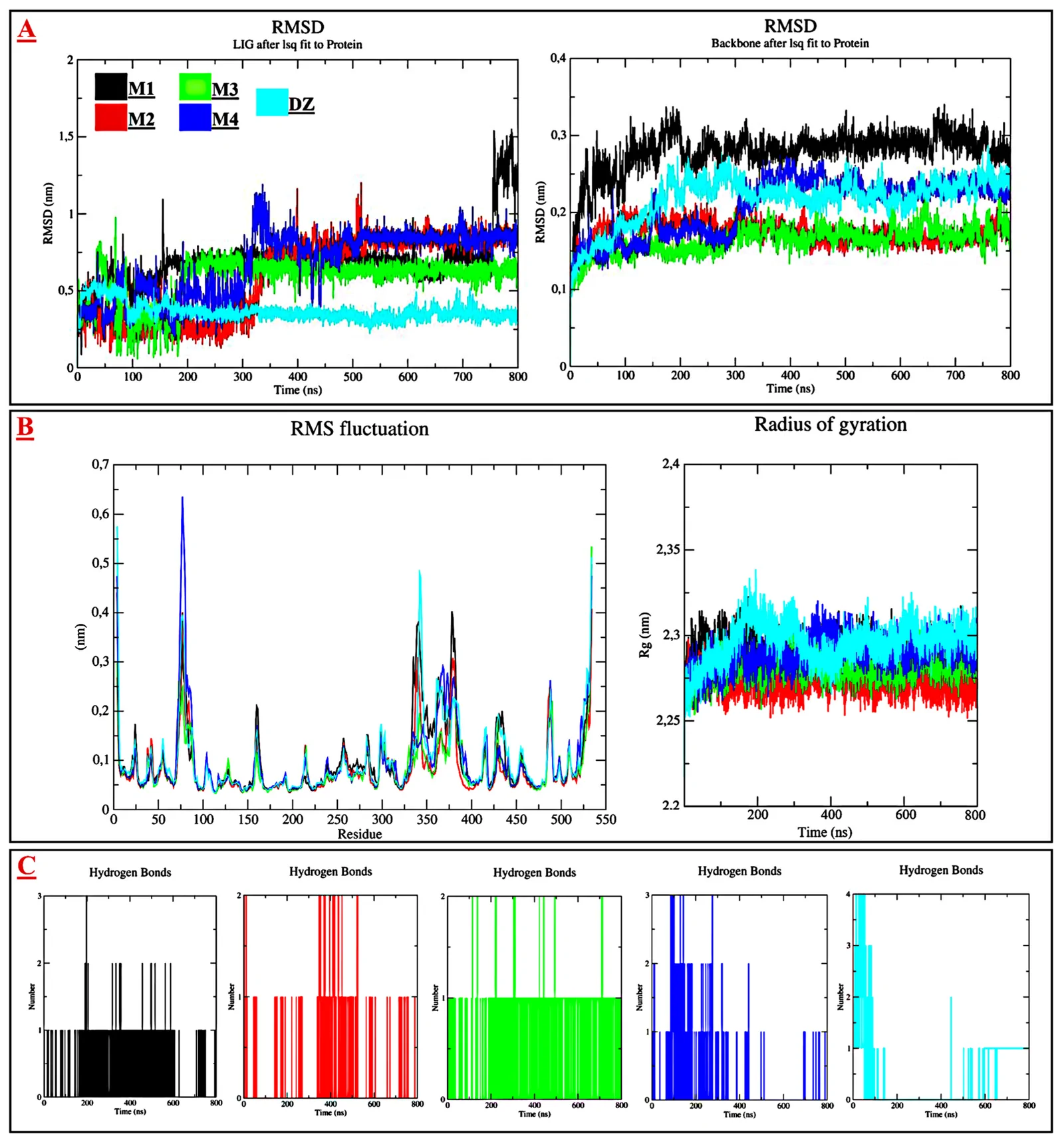
Long-timescale structural and interaction dynamics of AChE complexes with M1–M4 and donepezil (Dz) over 800 ns. (**A**) Ligand RMSD after protein fitting and protein-backbone RMSD; (**B**) Cα RMSF and radius of gyration; (**C**) time-resolved protein–ligand hydrogen-bond counts.

Across the 800 ns trajectories, donepezil maintained the lowest and most compact ligand-RMSD band after early relaxation. M3 remained within a comparatively narrow intermediate band, M2 and M4 underwent stepwise reorganizations near the middle of the simulation, and M1 showed a pronounced terminal excursion after approximately 760 ns. Protein backbone RMSD and radius-of-gyration profiles remained bounded across the five systems. Hydrogen-bond traces showed recurrent or intermittent ligand–protein contacts, with M4 displaying a richer early network that diminished after conformational reorganization (Figure 13).

### 2.6. Terminal-Ensemble MM/PBSA Analysis

Within the common 775–800 ns window, mean MM/PBSA ΔG_bind_ estimates were −18.85 ± 2.87 kcal mol^−1^ for M1, −20.14 ± 2.79 for M2, −23.90 ± 2.31 for M3, −15.95 ± 3.86 for M4, and −41.00 ± 2.75 for donepezil. M3, therefore, had the most favorable mean estimate among the chromone derivatives in this sampled interval (Table 5).

**Figure 14:**
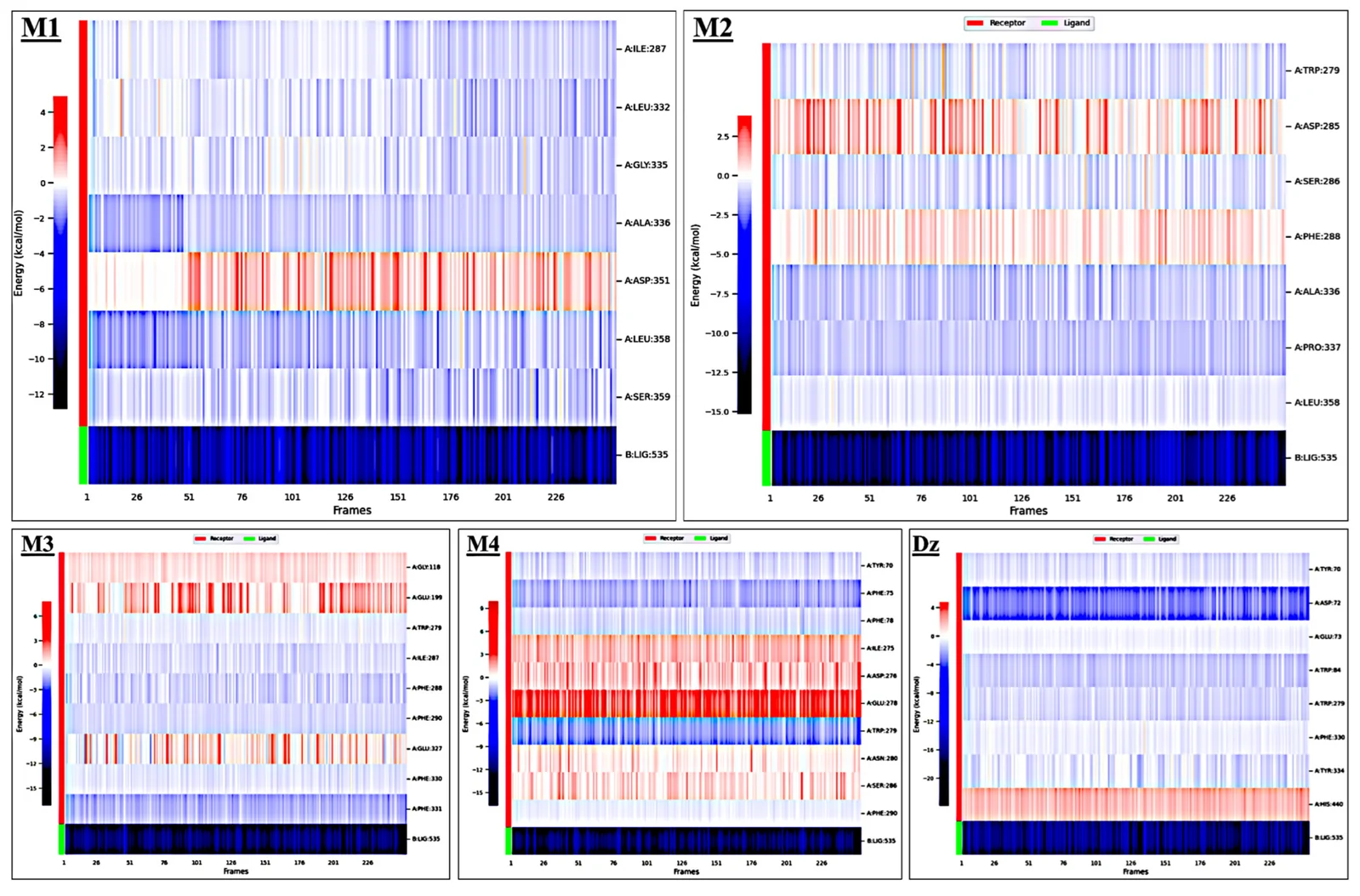
Residue-wise MM/PBSA energy-contribution maps for AChE complexes with M1–M4 and donepezil (Dz), based on 250 snapshots sampled every 100 ps over 775–800 ns. Blue and red regions denote favorable and unfavorable contributions, respectively.

The residue-wise MM/PBSA energy-contribution maps for the AChE complexes with M1–M4 and donepezil are presented in Figure 14. These maps show favorable and unfavorable residue contributions across the 250 snapshots sampled over the 775–800 ns interval.

### 2.7. PCA-Derived Free-Energy Landscapes and Dynamic Cross-Correlation

**Figure 15:**
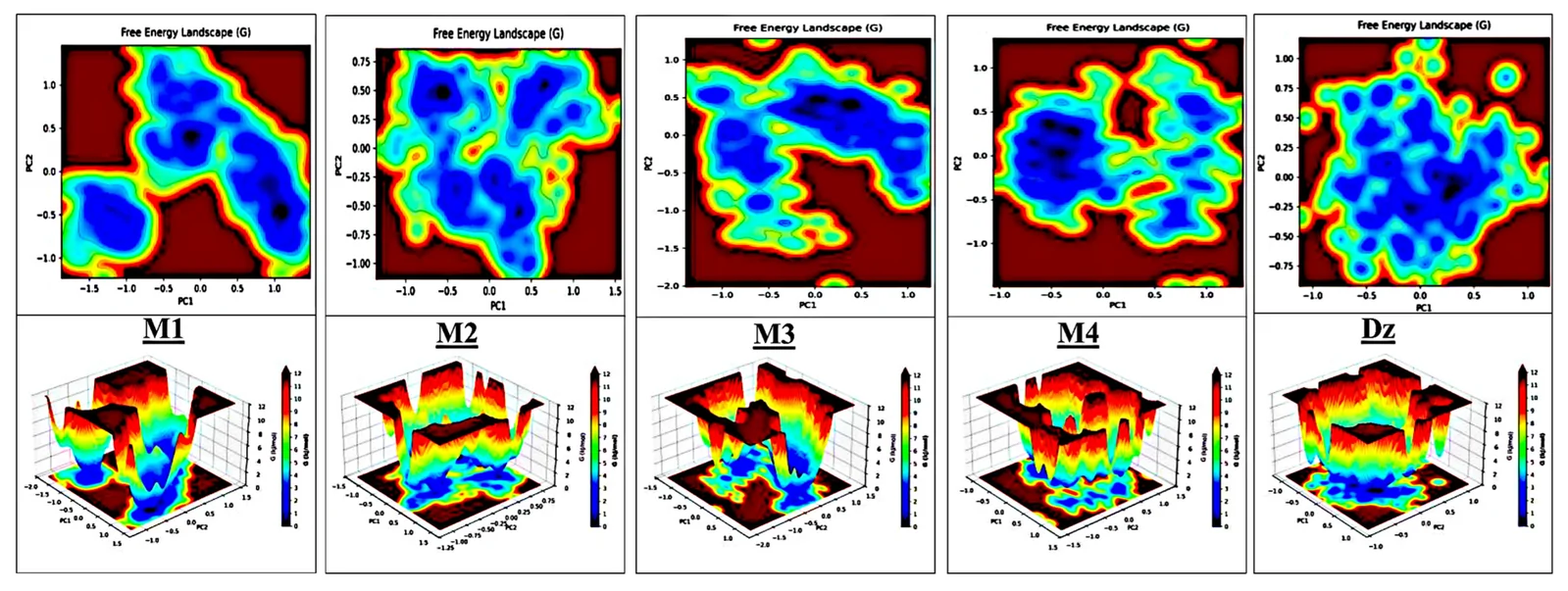
PCA-derived free-energy landscapes (2D PC1–PC2 contours and corresponding 3D surfaces) of AChE complexes with M1–M4 and donepezil (Dz), calculated over the terminal 775–800 ns interval. Blue regions indicate the most populated low-relative-free-energy states, whereas warmer colors denote progressively less populated states.

**Figure 16:**
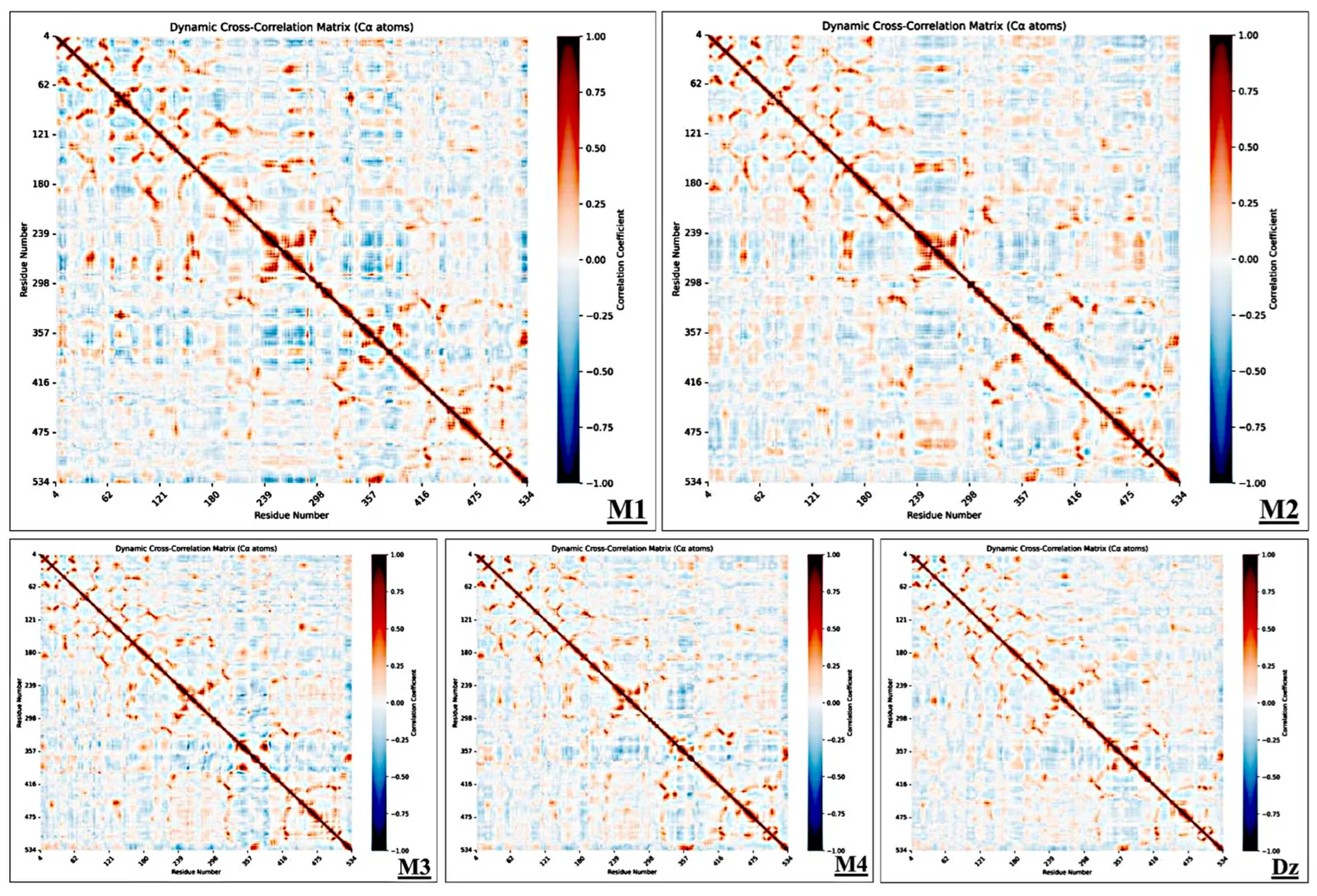
Dynamic cross-correlation matrices of Cα motions for AChE complexes with M1–M4 and donepezil (Dz), calculated over 775–800 ns. Red regions denote positively correlated motion, blue regions denote anticorrelated motion, and near-white regions denote weak correlation.

PCA/FEL analysis showed distinct terminal conformational landscapes among the complexes: M1 occupied three principal low-energy regions, M2 a broad interconnected multi-minimum basin, M3 a dominant elongated low-energy basin with connected subsidiary minima, M4 two major low-energy families, and donepezil an extensive central low-energy basin with several subminima (Figure 15). DCCM maps showed ligand-dependent off-diagonal correlation patterns, with particularly conspicuous alternating correlation/anticorrelation blocks for M2 and M4 (Figure 16).

### 2.8. Density Functional Theory: Electronic Stability and Reactivity

#### 2.8.1. Global Reactivity Descriptors and Frontier Molecular Orbitals

**Figure 17:**
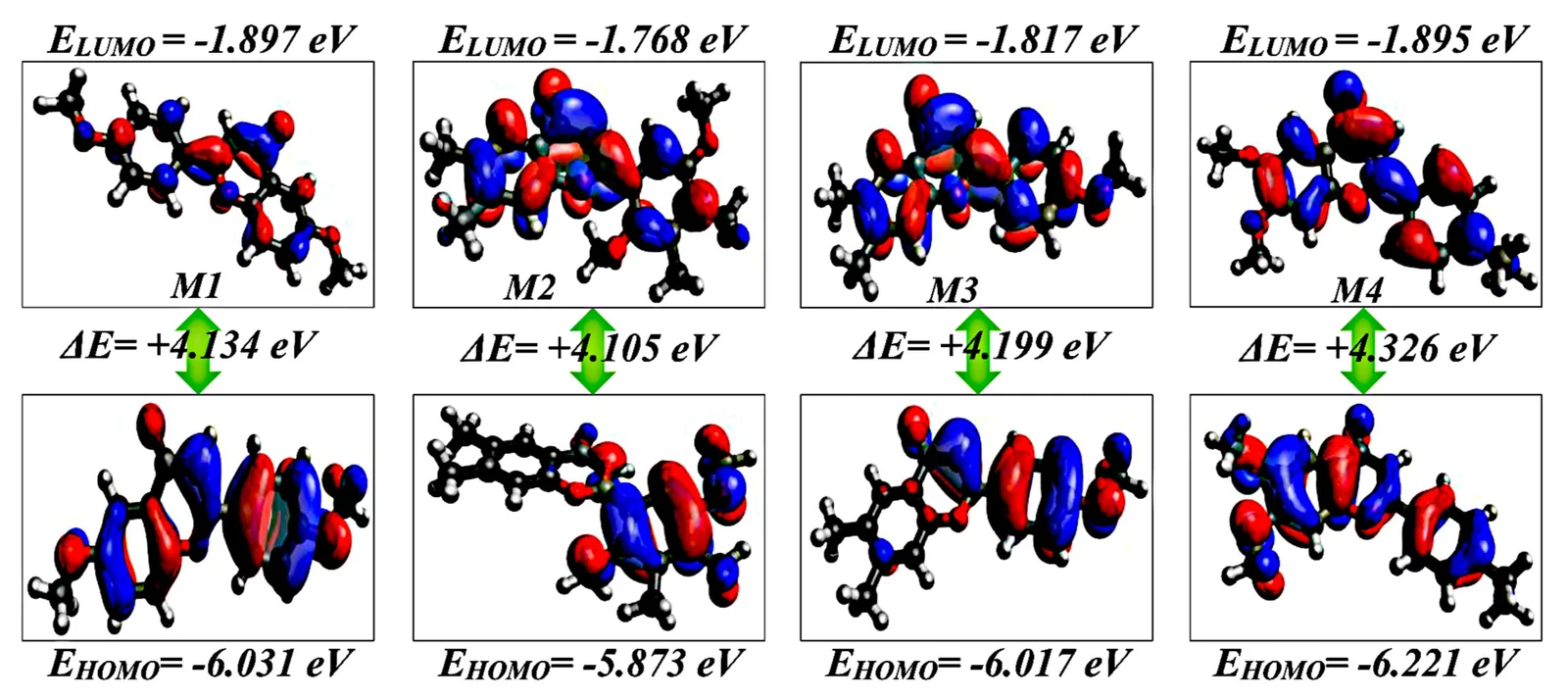
Frontier molecular orbitals (HOMO/LUMO isosurfaces) and HOMO–LUMO energy gaps (ΔE) for M1–M4.

M4 showed the lowest HOMO energy (−6.221 eV), the largest HOMO–LUMO gap (4.326 eV), the highest hardness (2.163 eV), and the lowest softness (0.462 eV^−1^). M2 showed the smallest energy gap (4.105 eV) and the greatest softness (0.487 eV^−1^). The complete global reactivity descriptors are presented in Table 6, and frontier-orbital distributions are shown in Figure 17.

#### 2.8.2. Molecular Electrostatic Potential and Electron Localization Function Analyses

**Figure 18:**
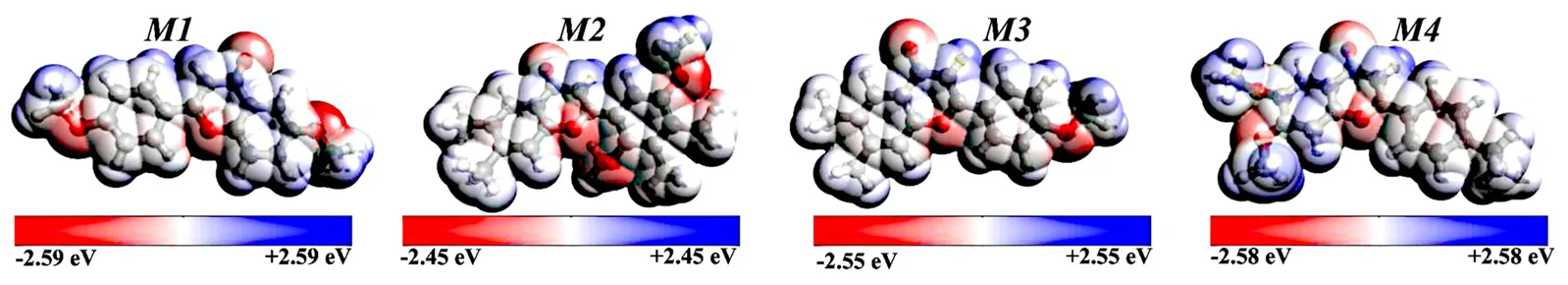
MEP surfaces of M1–M4 mapped onto electron density charts (red: negative/electron-rich; blue: positive/electron-poor).

**Figure 19:**
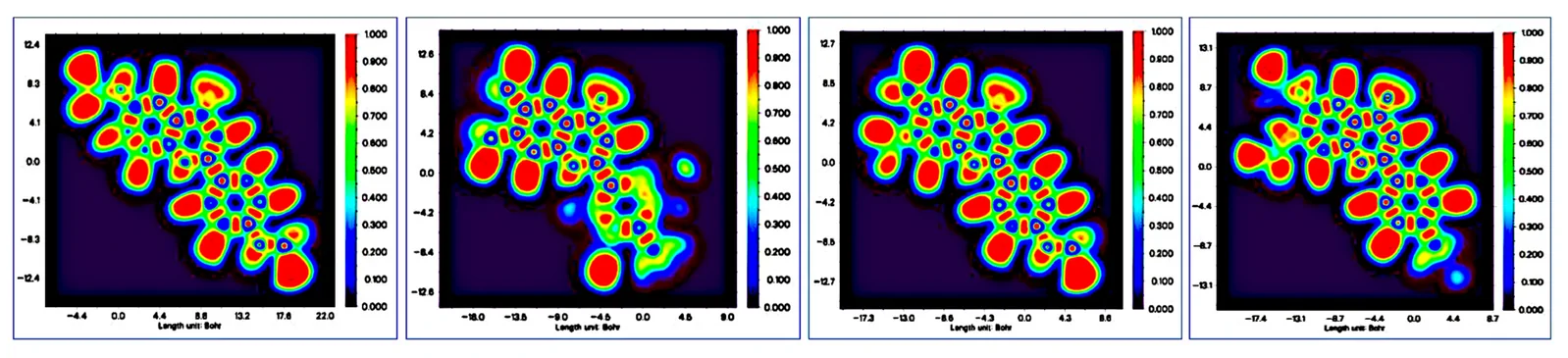
ELF maps of M1–M4 highlighting electron localization domains (0–1 scale).

MEP maps identified the most negative electrostatic regions around carbonyl and methoxy oxygen atoms across the series, while ELF maps showed high electron localization around heteroatoms and delocalized regions over the aromatic frameworks (Figure 18 and Figure 19).

#### 2.8.3. Noncovalent Interaction Analysis

**Figure 20:**
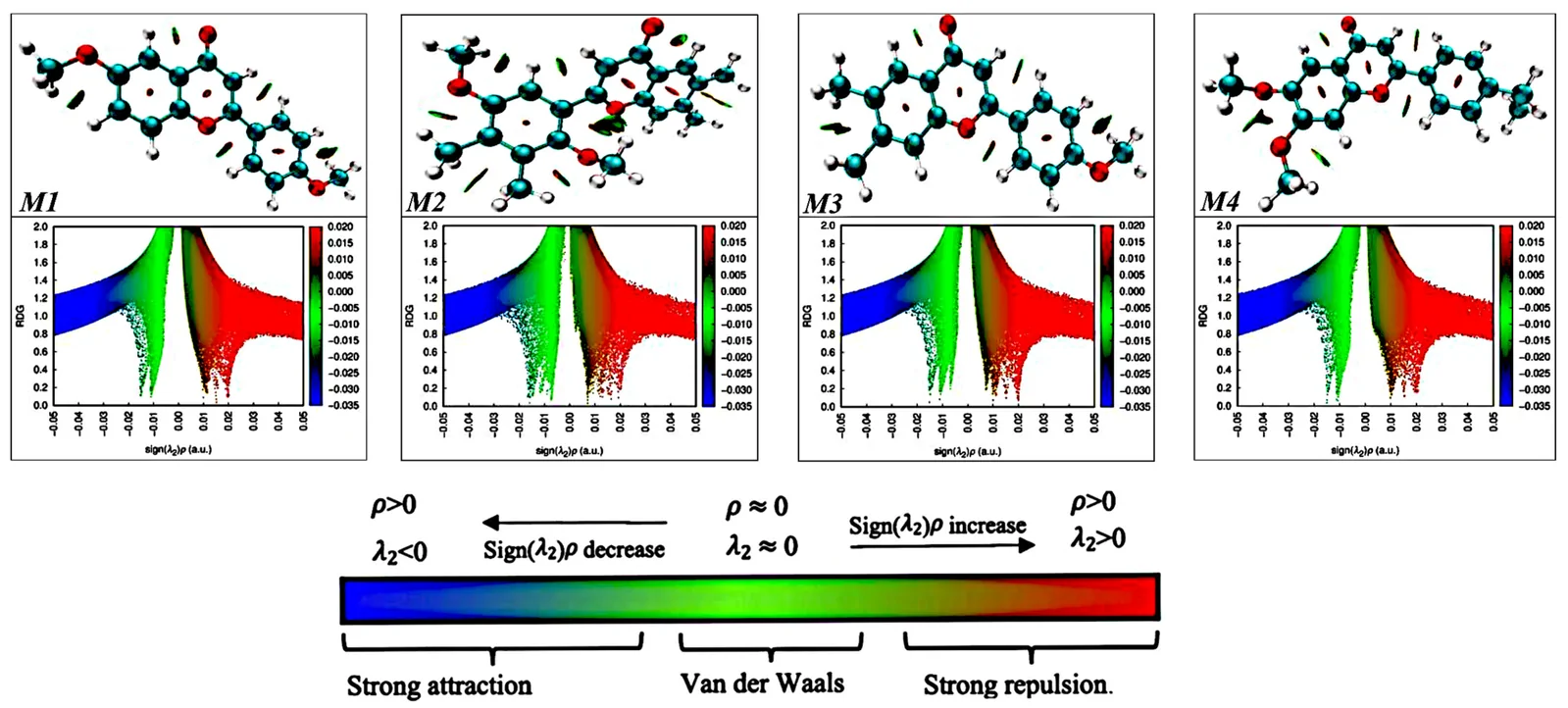
NCI isosurfaces and RDG versus sign(λ_2_)ρ plots for M1–M4 (blue: strong attraction; green: van der Waals force; red: strong repulsion).

NCI analysis showed that the chromone series was dominated by van der Waals/dispersive regions, with compound-dependent differences in the balance between dispersion and steric repulsion. M4 displayed extensive dispersive regions with comparatively limited repulsive congestion, whereas M2 showed greater local steric competition (Figure 20).

## 3. Discussion

### 3.1. ADME Profile and Pharmacokinetic Implications

The bioavailability-radar plots generated by SwissADME (Figure 5) indicate that the chromone derivatives occupy a broadly drug-like predicted physicochemical region that partly overlaps that of the reference inhibitor, while displaying nuanced divergences that can be rationalized at the structural level. Their highly conjugated bicyclic scaffold imposes intrinsic rigidity and a low fraction sp^3^, which explains why all four radar results contract along the saturation axis; donepezil, by contrast, contains a partially saturated indanone nucleus and an aliphatic piperidine linker that restore the required degree of three-dimensionality, thereby filling the INSATU sector more completely. Lipophilicity follows the electron-donating pattern of the substituents on the B-ring: the single para-methoxy group of M1 produces the least extended lipophilic wedge; the sequential addition of methyl and methoxy groups in M2 broadens this sector markedly, whereas the dimethyl (M3) and ethyl (M4) substitutions generate intermediate profiles that converge toward the balanced hydrophobic signature of donepezil [16]. The size and polarity sectors remain well-centered for all chromones, which is a direct consequence of their moderate molecular mass and of the limited number of heteroatoms confined to carbonyl and methoxy oxygen groups; donepezil attains comparable polarity thanks to its dimethoxy indanone, although the tertiary amine introduces a basic site that is absent in the flavones. Flexibility is minimal for the chromone series because torsional motion is largely restricted to the biaryl bond, whereas the piperidinyl–benzyl arm of donepezil inflates this spoke, reflecting higher conformational freedom, a property that may facilitate adaptive binding but at the cost of entropic penalties. Finally, the solubility spokes of the flavones remain within the ideal perimeter, suggesting a model-dependent balance between polarity and hydrophobic surface area, while the reference compound shows a slightly more peripheral trace, consistent with its bulkier hydrophobic domain. Collectively, the radar results provide a descriptive comparison of physicochemical properties; the low saturation index remains a deviation rather than a feature demonstrated to be compensated for by the other axes. These profiles do not establish superior brain penetration, exposure, or pharmacokinetic efficiency relative to the results for donepezil [23].

A holistic examination of the physicochemical descriptors reveals that the four flavone candidates occupy a “drug-like” space overlapping that of the reference donepezil (Table 1). Molecular weights for M1 (282 g mol^−1^) and M3 (280 g mol^−1^) lie well below the ∼450 g mol^−1^ ceiling generally associated with efficient blood–brain barrier (BBB) diffusion, while M2 (338 g mol^−1^) and M4 (310 g mol^−1^) remain substantially lighter than donepezil (379 g mol^−1^). Rotational freedom is likewise restricted (2–4 rotatable bonds versus six in donepezil), indicating lower formal flexibility, without quantifying the entropy of permeation or binding. The parent structures carry three to four hydrogen-bond acceptors and no hydrogen-bond donors in these ADME inputs, producing moderate polarity without sacrificing anchoring potential through carbonyl and ether oxygens. This balance is mirrored by topological polar surface areas of 39–49 Å^2^, all comfortably beneath the 90 Å^2^ BBB threshold and matching donepezil’s ≈ 39 Å^2^. Lipophilicity levels (cLogP = 3.1–4.4) cluster tightly around donepezil’s value (4.0): these values describe calculated lipophilicity rather than measured solubility, permeability, or plasma–protein binding. The chromone derivatives showed no violations of Lipinski’s or Veber’s criteria and shared a predicted bioavailability score of 0.55 [16]. Collectively, the lower mass, reduced flexibility and modest TPSA motivate experimental permeability and disposition testing, but cannot establish enhanced cerebral penetration, systemic stability, or oral availability relative to the results for donepezil [24].

The BOILED-Egg plot (Figure 6) shows that all four chromone derivatives occupy both the white ellipse of high gastrointestinal absorption and the yellow circle denoting passive blood–brain barrier permeability, indicating a lipophilicity/polarity balance comparable to that of the reference drug. Their upward shift on the W LOG P axis mirrors the incremental hydrophobicity conferred by their substituents, but plot membership does not demonstrate avoidance of membrane trapping, while the modest polar surface area intrinsic to the flavone scaffold keeps them centered in the yellow region. Donepezil’s plot point nearly coincides with that of M3, indicating its proximity in descriptor space rather than an independent validation of brain exposure. Color coding, however, reveals a crucial pharmacokinetic distinction: only donepezil, plotted in blue, is predicted to be a P-glycoprotein substrate (P-gp+), whereas all flavones, M1, M2, M3 and M4, plotted in red, are predicted non-substrates (P-gp−). These P-gp classifications are model predictions, not transport measurements. They do not establish the structural cause of transporter recognition nor the greater brain retention of M1–M4 at equivalent dosing. Passive permeability, efflux, unbound exposure, and metabolism require separate experimental characterization [25].

#### 3.1.1. Absorption, Distribution and Brain Penetration

The quantitative pharmacokinetic outputs in Figure 7 provide a computational comparison of absorption and distribution properties for all the chromone derivatives and for donepezil. Predicted intestinal absorption exceeds 97% for M1–M4, compared with approximately 93% for donepezil, which contains a protonatable tertiary amine linked to a flexible benzyl–piperidine arm. Predicted LogBB values show modest differences: M3 reaches 0.33, close to the levels for donepezil (0.34), compared with 0.20 for M1 and 0.26 for both M2 and M4 [17]. Methoxy enrichment (M4) or methoxy/methyl clustering on the B-ring (M2) yields intermediate permeability (0.26), consistent with differences in calculated descriptor inputs rather than indicative of a demonstrated transport mechanism. These subtleties are mirrored in logPS (CNS permeability): each chromone retains a value of around −1.4 log cm s^−1^, but this magnitude is not sufficient to establish adequate CNS exposure; the slightly less negative score of M3 (−1.39) does not quantify a reduction in desolvation penalties [26,27]. These outputs provide a comparative description reflecting differences in molecular structures and the physicochemical descriptors supplied to the model. CNS permeability assessment is complemented by logPS values of approximately −1.4 across the chromone series, with particularly similar estimates for M3 (−1.39) and donepezil (−1.41) [28,29]. This proximity indicates comparable predicted profiles within the model. Such descriptor-based assessment is consistent with drug-likeness and ADMET screening approaches also used to prioritize indolizine derivatives [30].

A more pronounced difference emerges in regards to predicted distribution volume: donepezil has a logVdss of 1.26, whereas the chromone derivatives range from 0.18 to 0.46. Within the series, M2 shows the highest value (0.463), and M4 the lowest (0.183), identifying predicted distribution differences for experimental investigation. Together, intestinal absorption, LogBB, logPS, and logVdss estimates provide a basis for prioritizing permeability and disposition experiments. Establishing how these differences translate into brain exposure, efflux, and tissue accumulation requires experimental validation.

#### 3.1.2. Metabolism, Transport and Clearance

The pkCSM outputs in Table 2 provide a comparative computational profile of metabolism, transport, and excretion. M1–M4 are predicted to be neither substrates nor inhibitors of CYP2D6, whereas donepezil is classified as an inhibitor, with a negative substrate classification. All five compounds are predicted to be CYP3A4 substrates, while only donepezil receives a positive CYP3A4-inhibitor classification [17]. These outputs identify priorities for enzyme assays, including substrate characterization and determination of inhibition potency and mechanism. Reaction-phenotyping and enzyme-kinetic studies of β-eudesmol involving CYP2C19 and CYP3A4 illustrate how these relationships can be characterized experimentally [28].

The platform also classifies all five compounds as substrates of the renal organic cation transporter OCT2, identifying a shared potential transport pathway for investigation. Evaluating differences in transport rates, tubular secretion, and renal clearance requires transporter-specific functional measurements [29].

Estimated total-clearance values range from log CL = 0.45–0.68 for the chromone derivatives, compared with approximately 1.0 for donepezil, with CL expressed in mL min^−1^ kg^−1^. These values are interpreted separately from CYP-inhibition classifications, since reduced enzyme activity may decrease the contribution of the corresponding metabolic pathway to substrate clearance. Accordingly, the analysis distinguishes substrate status, enzyme inhibition, transport, and total clearance, providing a basis for experimental investigation of pharmacokinetic differences between the chromone derivatives and donepezil and assessment of potential drug–drug interactions.

### 3.2. Interpretation of ProTox-III Predictions

Figure 8 and Table 3 were interpreted at two complementary levels. Figure 8 summarizes predicted acute oral hazard, together with the local applicability of the model, whereas Table 3 resolves endpoint-specific organ, pathway, receptor, stress-response, and metabolic alerts. These outputs do not constitute measured toxicity and should not be reduced to a single safe/unsafe label classification; predicted class, endpoint status, probability, structural similarity, and model accuracy must be considered together.

Figure 8 differentiates the chromone series from the reference comparator at the acute-hazard level. M1–M4 were uniformly assigned a predicted LD_50_ of 4000 mg kg^−1^ and GHS class 5, whereas Dz was assigned 505 mg kg^−1^ and class 4. Within this model, the chromones therefore occupy a less severe acute oral toxicity category than that for Dz. The applicability metrics, however, are compound-dependent. M1 has the strongest support, combining 95.18% average similarity with 72.90% reported prediction accuracy. M4 follows, with 80.03% similarity and 70.97% accuracy, while M3 shows results of 78.37% and 69.26%, respectively. M2 has the lowest similarity (64.00%) and an accuracy estimate of 68.07%, so its class assignment carries the greatest extrapolation uncertainty within the series [18,31]. These metrics describe proximity to the model’s reference domain and expected predictive performance; they do not measure the chemical, mechanistic, or clinical similarity to Dz. Accordingly, the acute-toxicity outputs were used only for comparative prioritization and for defining confirmatory assays, not as evidence of clinical safety.

Table 3 reveals a substituent-dependent liability pattern rather than a common chromone-class signature. At the organ level, all four derivatives were predicted to be inactive for hepatotoxicity and cardiotoxicity, but each carried low-to-moderate-confidence nephrotoxicity and respiratory-toxicity alerts. Dz showed the opposite renal pattern but a high-probability neurotoxicity signal (90%) and a very strong respiratory-toxicity signal (96%). Thus, the apparently favorable acute class of M1–M4 in Figure 8 does not eliminate organ-specific concerns; acute lethality and mechanistic toxicity are distinct prediction layers.

The pathway-level profile identifies the most important experimental priorities. AhR activation was predicted for every chromone, and M1–M3 also carried estrogen-receptor-alpha alerts, whereas M4 was predicted to be inactive for this endpoint. Potential mitochondrial-membrane disruption was flagged for M2–M4, while ATAD5 activation was predicted for M1–M3 but not M4. All four derivatives were predicted to be active toward transthyretin and GABA-receptor endpoints, although the GABA probabilities were modest, while NMDA, AMPA, kainate, voltage-gated sodium-channel, and thyroid-receptor endpoints were consistently inactive, with generally higher confidence. The AChE target calls should not be interpreted as an activity ranking: the high-confidence Dz signal and the weak or inactive chromone calls originate from a toxicity-classification model and do not replace enzyme-inhibition measurements or the structure-based analyses.

Metabolic alerts further differentiate the candidates. CYP1A2, CYP2C19, and CYP2C9 activity were predicted across most or all chromones, with M1 additionally showing a strong CYP3A4 signal; CYP2D6 and CYP2E1 were consistently inactive for M1–M4. Among the designed compounds, M4 has the fewest predicted active classifications for carcinogenicity, immunotoxicity, cytotoxicity, ER-alpha signaling, and ATAD5 among the chromones, but still retains renal, respiratory, AhR, mitochondrial, transthyretin, GABA, and CYP alerts. M3 combines the most favorable MM/PBSA estimate with several safety flags, including low-confidence carcinogenicity and stronger AhR/ATAD5/TTR signals. The integrated interpretation therefore prioritizes M3 for testing the terminal-window energetic hypothesis and M4 for testing a complementary electronic/toxicity-profile hypothesis, with both requiring dose–response cytotoxicity, genotoxicity, mitochondrial-function, renal, respiratory, endocrine-receptor, and CYP interaction assays before drawing any safety conclusion.

### 3.3. Molecular Docking

The docking calculations used the *Torpedo californica* acetylcholinesterase structure 7B2W. The 2.65 Å X-ray model (Rwork/Rfree = 0.161/0.212) provides an experimentally determined non-human receptor framework for examining local ligand accommodation within the AChE gorge. Human 4EY7 provided the crystallographic donepezil reference for redocking and structural transfer into the 7B2W coordinate frame (Figure 4). The resulting 0.942 Å redocking RMSD and 1.074 Å cross-docking pose RMSD support recovery of a donepezil reference orientation. The refined protein Cα RMSD of 0.795 Å quantifies structural agreement over the retained aligned atoms, providing context for the reference-pose transfer. The focused M1–M4 docking results are consequently interpreted as structural hypotheses in 7B2W, supporting analysis of local recognition patterns under the selected docking conditions and guiding subsequent computational and experimental assessment [32].

The docking results in Table 4 are AutoDock Vina scoring-function estimates used to rank candidate binding poses and are interpreted as comparative computational scores. Applying identical receptor preparation, grid dimensions, and search settings across M1–M4 and donepezil provides a common framework for comparing their predicted binding modes within the selected region [33]. M4 produced the most negative Vina score (−9.6 kcal mol^−1^), followed closely by M2 (−9.5 kcal mol^−1^), donepezil (Dz; −9.3 kcal mol^−1^), M3 (−9.2 kcal mol^−1^), and M1 (−9.0 kcal mol^−1^). M4 combines the reported multipoint polar-contact pattern with extensive aromatic and hydrophobic complementarity, whereas M2 and M3 are dominated by nonpolar packing. M1 and Dz retain discrete polar anchors but differ in regards to scaffold rigidity and conformational freedom. Because the score differences are small and the Vina function does not explicitly resolve full solvent reorganization, conformational entropy, or long-timescale pose adaptation, this ranking was used to select and rationalize docked orientations, not to claim quantitative affinity. The subsequent 800 ns trajectories and MM/PBSA analysis provide a complementary, sampling-dependent comparison rather than an independent validation of experimental affinity.

Figure 9 depicts M1 lodged in the 7B2W pocket through two T-shaped π-π contacts with Trp84 (5.16 Å) and Tyr131 (5.32 Å), a π-alkyl interaction with Trp84 (4.53 Å), and nine surrounding van der Waals contacts. The SAS surface is predominantly green, signifying burial in a hydrophobic cleft. Hydrogen-bond donor/acceptor mapping shows minimal magenta or green regions, indicating scarce polar contacts. The aromatic edge/face surface highlights the blue edge zones of M1 aligned toward the aromatic residues involved in stacking. The interpolated electrostatic surface is largely neutral to slightly negative, matching the apolar environment of the binding site. Relative to M1, compound M2 embeds in the 7B2W cavity through a denser aromatic network (Figure 10), exhibiting five π-π contact dual T-shaped/stacked interactions with Trp279 at 4.54 Å and 5.38 Å, and single contacts with Tyr121, Phe330, and Tyr334 spanning 4.46–5.68 Å complemented by a π-σ interaction to Trp279 at 3.71 Å, ten π–alkyl contacts in the 4.23–5.42 Å range, and seven surrounding van der Waals contacts. The SAS map alternates green and deep-blue zones, indicating that one face of M2 remains buried while another projects modestly toward a solvent geometry, contrasting with the uniformly buried profile of M1. On the hydrogen-bond donor/acceptor surface, fewer magenta and green patches appear around the ligand’s periphery, indicating fewer potential polar anchoring points than observed in M1. The aromatic edge/face surface reveals an interwoven mosaic of blue edge and orange face regions, reflecting the coexistence of T-shaped and stacked geometries and highlighting a richer π-system engagement than observed previously. The interpolated charge surface stays largely neutral-gray with faint negative islands, maintaining compatibility with the hydrophobic pocket while introducing localized electrostatic complementarity around Trp279 [33,34].

As illustrated in Figure 11, M3 secures a balanced pose: three face-to-face π-stacked contacts, two with Trp279 (4.35 Å, 4.66 Å) and one with Tyr334 (4.36 Å), are augmented by three alkyl/π–alkyl interactions (Leu282 5.08 Å, Phe330 5.25 Å, Trp279 5.03 Å) and ten van der Waals contacts. Ref. [35] Compared with M1’s deeply buried orientation and M2’s more solvent-exposed geometry, M3 occupies an intermediate depth, as indicated by the weaker green SAS band and broader blue regions shown in Figure 11. Sparse but strategically positioned donor/acceptor regions provide limited polar anchoring, while the aromatic surface map confirms the predominance of π-stacking. The largely neutral interpolated-charge surface, interrupted only by small negative regions, is compatible with a recognition mode driven mainly by aromatic packing and dispersion rather than by an extensive hydrogen-bond network [36].

Although M4 does not exhibit the highest predicted BBB permeability, it remains within the modeled CNS-accessible region and has the smallest predicted total clearance in the series. Its class 5 acute-toxicity prediction is also less severe than the class 4 prediction for Dz. Most importantly for the docking stage, M4 produced the most negative Vina score and the richest reported polar-contact pattern, which justified the detailed structural visualization in Figure 12. This selection should not be interpreted as an overall lead declaration: the later MD and MM/PBSA analyses explicitly test whether the contact-rich docked pose persists after solvent relaxation and conformational reorganization.

The convergence of the 2D interaction map and 3D surface analyses in Figure 12 identifies the docking-level basis of M4 complementarity. The reported contact geometry includes two shorter conventional hydrogen-bond arrangements involving Gly118 and Tyr121 (2.49 and 2.35 Å), accompanied by longer carbon–hydrogen contacts involving Gly117 and Glu199 (3.77 and 3.72 Å). Together, these interactions position the oxygen-rich chromone region toward the polar/oxyanion environment while retaining an extended aromatic surface within the gorge. This multipoint arrangement supports a local gorge-accommodation hypothesis, but a static docking map cannot establish contact occupancy or solution-phase persistence [33,37].

Hydrophobic and aromatic contacts further define the docked orientation. The chromone core engages Trp84 and Phe330 through face-to-face or offset π contacts, while an edge-to-face interaction with Tyr334 and additional π–alkyl contacts enlarge the nonpolar contact surface. The surrounding van der Waals shell is consistent with closed shape complementarity and reduced orientational freedom in the selected pose. These geometric features rationalize the docking score, but their energetic persistence must be evaluated from the explicit-solvent trajectory rather than inferred from the static contact count.

The 3-D surfaces corroborate this complementarity: the SAS map shows near-total burial (10–18 Å^2^) inside the pocket; the H-bond donor/acceptor map aligns the carbonyl-methoxy acceptor patch with the donor band of Gly118 and Tyr121; and the interpolated-charge surface illustrates a negative carbonyl cloud nesting in the positive trench created by the Gly117/118 loop. The aromatic edge/face map reveals orange “face” regions of the chromone stacked parallel to blue “edge” rims of Trp84 and Phe330, while a second blue edge from Tyr334 sustains the T-shaped geometry, complemented by the π–alkyl contacts.

Taken together, Figure 12 supports a contact-rich local docking hypothesis for M4, combining polar orientation with aromatic and hydrophobic complementarity. Docking alone does not quantify configurational entropy or solution-phase affinity, and the apparent multiplicity of contacts should not be equated with persistent occupancy. The later MD/MM/PBSA results are therefore essential: they show that M4’s favorable docking geometry does not translate into the most favorable terminal-window end-state energy. Enzymatic inhibition, kinetic mechanism, and any effect on Aβ assembly remain experimental questions.

The distances annotated in Figure 12 must be interpreted according to contact class and geometry. Aromatic and hydrophobic annotations extending beyond 5 Å are not conventional hydrogen bonds and are not treated as evidence of strong individual stabilization. Their interpretation depends on the atoms or ring features used to define the distance, ring orientation, and the surrounding contact geometry. The map alone does not establish energetic significance or persistence. Multiple residues can contact different faces or substituents of the same ligand without competing for a single bond; mutually incompatible contacts would require alternative geometries. Neither contact priority nor relative occupancy can be assigned from a static diagram or from the shortest distance alone.

### 3.4. Long-Timescale Molecular Dynamics

The 800 ns simulations were analyzed as ensemble trajectories rather than as a search for an invariant continuation of the docking pose. Ligand RMSD was calculated after fitting the protein and therefore reports movement of the ligand relative to the enzyme; stepwise changes can represent reorientation within the gorge and should not be equated automatically with dissociation. Protein-backbone RMSD, RMSF, radius of gyration (Rg), and hydrogen-bond occupancy were interpreted jointly to distinguish local adaptation from global destabilization [38,39,40].

Figure 13A shows that every protein backbone reaches a bounded regime, but the ligand coordinates follow distinct kinetic pathways. Dz maintains the lowest and most compact ligand-RMSD band after early relaxation, consistent with persistent localization in the gorge. M3 fluctuates within a comparatively narrow intermediate band throughout most of the trajectory and does not undergo a major late transition. M2 and M4 display clear stepwise reorganizations near the middle of the simulation, after which both occupy higher but stable RMSD plateaus; these trajectories are more consistent with conversion to alternative bound substates than with continuous drift [41,42]. M1 remains comparatively stable over most of the trajectory but undergoes a pronounced terminal excursion after approximately 760 ns. Consequently, the 775–800 ns MM/PBSA window represents a newly established late M1 geometry rather than the original docked orientation, an important distinction when comparing docking and end-state energetics.

At the protein level, M2 and M3 maintain the lowest backbone deviations, M4 and Dz occupy intermediate plateaus, and M1 shows the largest, yet still bounded, displacement. The absence of sustained growth in Rg across all five systems indicates preservation of global compactness and argues against ligand-induced unfolding. RMSF peaks are confined to a limited number of flexible segments and terminal regions, whereas most residues remain comparatively restrained. The stronger local peak observed for the M4 complex and the enhanced mobility in selected regions of M1 and Dz, therefore, reflect ligand-specific loop accommodation rather than a global loss of AChE architecture.

The hydrogen-bond traces provide a complementary but nonexclusive view of recognition. M3 retains a recurrent single hydrogen bond over much of the trajectory, whereas M1 and M2 show intermittent contacts with occasional short-lived increases in multiplicity. M4 exhibits the richest early hydrogen-bond network but loses most of these contacts after its conformational transition, demonstrating that the four-point docking network is not continuously preserved in solution. Dz similarly begins with several hydrogen bonds, followed by a long interval dominated by non-hydrogen-bond interactions and partial late recovery. Because the chromone core is largely aromatic and hydrophobic, the absence of a continuous hydrogen bond does not imply loss of binding; the stable Rg/backbone profiles and the favorable van der Waals terms shown in Table 5 indicate that dispersion, aromatic packing, and shape complementarity remain major stabilizing forces. Overall, Figure 13 supports persistent but dynamically distinct bound ensembles within the sampled trajectories while cautioning against treating the docking pose as the unique solution conformation.

### 3.5. Terminal-Ensemble MM/PBSA

MM/PBSA was used to compare the nonbonded and solvation balance of the common 775–800 ns ensemble. Because configurational entropy was not included and the snapshots are temporally correlated, the values are best interpreted as descriptive, within-protocol end-state estimates rather than as absolute experimental affinities or formal tests of small rank differences. The mean quantifies the central energetic tendency of the terminal ensemble, whereas the standard deviation reports its conformational sensitivity [43].

In Table 5, all five complexes have negative mean ΔG_bind_ estimates within the selected terminal window; this sign alone is not an experimental demonstration of binding. Dz is the most favorable system (−41.00 ± 2.75 kcal mol^−1^), followed within the chromone series by M3 (−23.90 ± 2.31), M2 (−20.14 ± 2.79), M1 (−18.85 ± 2.87), and M4 (−15.95 ± 3.86 kcal mol^−1^). The chromone ordering is therefore M3 > M2 > M1 > M4 under this specific protocol, but it is a window-specific ordering of mean estimates, not a temporally reproduced ranking or a quantitative potency scale.

The 775–800 ns interval samples M1 after its late rearrangement and does not establish that the same ranking would hold before that transition or in other late windows. The reported standard deviations quantify within-window dispersion, not independent replicate uncertainty or convergence. Thus, the numerical means are retained descriptively, but robustness of the ordering to the sampling window remains untested.

Energy-component analysis explains why docking and MM/PBSA produce different rankings. Van der Waals interactions are strongly favorable for every complex and increase from M1 to M4 within the chromone series, confirming that aromatic packing and hydrophobic complementarity are the common energetic foundation. M3 becomes the most favorable chromone because its markedly stronger electrostatic term (−14.79 kcal mol^−1^) provides sufficient additional stabilization to offset its sizable PB desolvation penalty. M2 achieves a balanced profile, with strong dispersion and a more moderate electrostatic contribution. M1 has weaker dispersion but also a smaller polar-solvation cost. M4 shows the most favorable chromone van der Waals and surface terms. Yet, these gains are more than offset by the largest PB penalty, explaining why its extensive docking contact network does not yield the best solution-phase end-state energy. This directly illustrates how solvent reorganization and electrostatic desolvation can reshape a static docking picture.

Protonated Dz (+1) operates in a different energetic regime. Its favorable electrostatic term (−264.24 ± 7.27 kcal mol^−1^) is nearly counterbalanced by the unfavorable PB contribution (273.53 ± 7.22 kcal mol^−1^); their sum yields an unfavorable net contribution of +9.29 kcal mol^−1^. The overall MM/PBSA estimate remains strongly favorable because the most favorable van der Waals and nonpolar terms outweigh this electrostatic–polar solvation penalty. These mutually compensating magnitudes should not be interpreted independently because changes in the charged/polar environment affect both terms simultaneously. The standard deviations describe energetic variability within the selected window: M3 has the smallest within-window standard deviation among the chromone estimates, whereas M4 shows the largest energetic spread, qualitatively consistent with its separated FEL basins and its post-transition hydrogen-bond reorganization.

Figure 14 resolves how the ensemble totals are distributed along the AChE gorge. Across the five maps, persistent favorable bands are concentrated in aromatic and hydrophobic regions that can sustain π-stacking and dispersion, while recurrent positive bands mark local electrostatic or desolvation penalties. M1 and M2 display stabilization dominated by a limited set of hydrophobic/aromatic positions, with isolated unfavorable acidic or polar contributions. M3 shows a broader and more persistent favorable network across the central and lower gorge, consistent with the cooperative electrostatic–dispersion balance that produces the best chromone ΔG_bind_. M4 makes strong favorable aromatic contributions but also exhibits a conspicuous, persistent unfavorable polar region, providing a local energetic counterpart to its large ΔG_PB_ penalty. Dz shows distributed stabilization across canonical aromatic-gorge positions, together with a localized unfavorable contribution near the catalytic region.

### 3.6. PCA/FEL and Dynamic Cross-Correlation

PCA/FEL and DCCM interrogate complementary layers of the terminal ensemble that are not captured by RMSD alone. PCA projects correlated Cα fluctuations onto dominant collective coordinates, the free-energy landscape maps the occupancy of these coordinates as relative free-energy basins, and DCCM identifies residue pairs that move in concert or in opposition [35,44]. These analyses describe conformational organization and communication; they do not measure ligand affinity or prove causality.

Each PCA/FEL and DCCM result is derived from one trajectory and the stated terminal interval. Differences between maps may depend on initial conditions and incomplete sampling; basin separation does not establish transition rates or a converged free-energy barrier, and cross-system dynamical differences have not been reproduced with independent seeds.

Figure 15 reveals a distinct energy-landscape topology for each ligand. The M1 complex occupies three principal low-energy regions connected through higher-energy corridors, describing heterogeneity within the terminal sample rather than establishing exchange kinetics or directly resolving the earlier ligand-RMSD transition. M2 exhibits a broad, interconnected, multi-minimum basin, consistent with adaptive motion within a continuously accessible conformational region rather than switching between sharply isolated states. M3 presents a dominant elongated low-energy basin with connected subsidiary minima, the most coherent landscape among the designed derivatives; this organization agrees with its narrow MM/PBSA dispersion and favorable terminal energy. M4 is partitioned into two major low-energy families separated by a pronounced higher-energy region, consistent with multiple sampled configurations, without demonstrating a converged interconversion mechanism. Dz displays an extensive central low-energy basin containing several closely spaced subminima, illustrating sampled conformational plasticity rather than independently establishing binding strength.

The 2D contours and 3D surfaces should be interpreted through basin number, connectivity, and occupied area, not by comparing the absolute height or color of isolated points. A deep basin denotes a frequently sampled region of the projected coordinates within the analyzed window, whereas connected minima do not by themselves establish metastability or low-barrier exchange; neither feature establishes a more favorable binding free energy across different ligands. The agreement among the M3 and M4 landscapes, the MM/PBSA standard deviations, and the ligand-RMSD transitions provides an internally consistent ensemble-level explanation for the smaller sampled energetic dispersion of M3 and the greater sampled heterogeneity of M4, without establishing reproducibility.

Figure 16 shows that all complexes retain strong short-range correlations along the diagonal, while their off-diagonal patterns differ. M1 contains moderate long-range positive and negative patches, consistent with a limited but detectable redistribution of motions during its late pose-change. M2 and M4 show the most conspicuous alternating correlation/anticorrelation blocks, indicating stronger coupling between separated regions and greater reorganization of collective motion. M3 displays a more distributed and balanced correlation pattern, without one dominant antagonistic block, consistent with a coherent but nonrigid terminal ensemble. Dz exhibits widespread, comparatively diffuse off-diagonal coupling, suggesting coordinated flexibility across the enzyme rather than uniform immobilization. The recurring coupling between distal regions and the central gorge is compatible with a hypothesis of ligand-associated covariance differences within AChE [35]; however, DCCM reports covariance, and directional allosteric causality would require network analysis and experimental validation.

### 3.7. Density Functional Theory

Conceptual DFT complements the structure-based analyses by describing intrinsic ligand electronics, but it does not validate or reproduce the MD/MM/PBSA ranking. Frontier-orbital energies, hardness, softness, chemical potential, and electrophilicity are isolated-molecule descriptors; protein affinity additionally depends on pose, solvation, conformational adaptation, and entropy [40,45].

M4 has the lowest HOMO energy (−6.221 eV), the largest HOMO–LUMO gap (4.326 eV), the highest hardness (2.163 eV), and the lowest softness (0.462 eV^−1^), identifying it as the chromone in this series that is the most electronically resistant to charge redistribution (Table 6). It also exhibits the most negative chemical potential (−4.058 eV) and the highest electrophilicity index (3.807 eV), although the latter is only marginally higher than that of M1 (3.801 eV). By contrast, M2 has the smallest gap (4.105 eV) and greatest softness (0.487 eV^−1^), consistent with higher polarizability and a greater propensity for electronic response. These trends characterize reactivity and stability, not stronger nor weaker binding by themselves.

HOMO and LUMO maps were included to describe how frontier-orbital density and the associated energy gap change across a shared scaffold with different substituents. Chemical inspection identifies likely carbonyl/ether acceptors, but does not supply these calculated orbital energies or spatial distributions. Frontier-orbital maps are distinct from molecular electrostatic potential maps: the latter describe the electrostatic potential, whereas the former describe occupied and unoccupied orbitals within the chosen electronic-structure approximation. The maps, therefore, complement the structural comparison, but are not essential evidence of AChE inhibition and do not independently predict binding affinity.

Table 6 shows that M4 combines the lowest HOMO energy (−6.221 eV) with the largest gap (ΔE ≈ 4.326 eV) and highest hardness (η ≈ 2.163), identifying the least polarizable scaffold in the series, while its electrophilicity index (ω ≈ 3.807) and chemical potential (μ ≈ −4.058 eV) describe an electron-accepting framework with well-defined oxygen-centered acceptor regions. M2, in contrast, has the highest HOMO energy (−5.873 eV), the smallest gap (ΔE ≈ 4.105 eV), and the greatest softness (S ≈ 0.487 eV^−1^), indicating a more readily polarizable electronic structure. Figure 17 confirms that the frontier orbitals remain extensively delocalized over the conjugated π framework, while substituent identity redistributes orbital density near the heteroatoms. These isolated-molecule descriptors rationalize possible differences in polarizability and interaction directionality but do not reproduce the MD/MM/PBSA binding ranking.

#### 3.7.1. Molecular Electrostatic Potential and Electron Localization Function

MEP identifies electrostatic hot spots (electron-rich versus electron-poor regions) that predict where H-bonding and electrostatic contacts are most likely to occur. ELF quantifies electron localization (lone pairs, bonding basins, π localization), describing spatial localization rather than directly measuring protein–ligand interaction strength [46,47].

In Figure 18, all ligands show the most negative MEP regions concentrated around the carbonyl and methoxy oxygens, confirming these atoms as primary H-bond acceptor sites; M4 exhibits well-positioned negative patches consistent with its ability to form multipoint polar anchoring in the gorge, without establishing the persistence or energetic contribution of these contacts in the protein. Figure 19 complements this by revealing high ELF localization around heteroatoms (lone-pair basins) and delocalized regions over the aromatic scaffold (π framework): M4 maintains both strong lone-pair localization (supporting robust acceptor character) and an extended π system (supporting stacking along the aromatic channel), features that motivate testing but do not demonstrate optimized or dual-site AChE inhibition.

#### 3.7.2. Noncovalent Interaction Analysis

NCI analysis provides a compact visualization of weak interactions (attractive, dispersive, repulsive) that govern intramolecular preorganization and therefore, influence how easily a ligand adopts a binding-competent conformation [48].

Figure 20 indicates that the chromone series is dominated by van der Waals/dispersive features, consistent with aromatic scaffolds whose intramolecular organization is governed mainly by weak packing interactions. The principal distinction is the balance between dispersion and steric repulsion. M4 displays extensive dispersive regions with comparatively limited repulsive congestion, supporting a well-preorganized and electronically rigid isolated-ligand geometry. The more heavily substituted M2 profile shows greater local steric competition, compatible with higher polarizability and broader conformational exploration. NCI maps describe intramolecular preorganization rather than protein-binding free energy; consequently, they do not override the MM/PBSA result. The integrated interpretation is that M4 is distinguished by electronic stability and favorable preorganization, whereas M3 has the most favorable mean chromone–AChE estimate in the single analyzed terminal window, with temporal reproducibility remaining untested.

## 4. Materials and Methods

### 4.1. Compound Selection and Pharmacokinetic Analysis Using Computational Tools

M1–M4 were designed in this study as a focused computational series, rather than selected from an experimentally screened library or isolated from a biological source. The shared 2-arylchromen-4-one framework provides a conjugated aromatic surface and carbonyl/ether acceptors. Methoxy, methyl, and ethyl substitution was varied to explore differences in polarity, hydrophobic surface, rigidity, and electronic distribution, while avoiding a readily protonatable amine or free phenolic acid–base site near physiological pH. The neutral forms of M1–M4 were therefore selected at pH 7.4; this fixed-state design does not imply invariance across all pH conditions. No synthesis, isolation, or preliminary enzyme-inhibition measurements are reported.

Structurally, M1 combines a 6-methoxy substituent with a para-methoxyphenyl group; M2 combines 6,7-dimethyl substitution with a dimethoxy/dimethyl-substituted phenyl ring; M3 retains the para-methoxyphenyl group with 6,7-dimethyl substitution; and M4 combines a para-ethylphenyl group with 6,7-dimethoxy substitution. No tetrahedral stereogenic center is present in the depicted M1–M4 structures. Donepezil, instead, contains a dimethoxyindanone, a flexible benzylpiperidine moiety, a protonatable tertiary amine, and an indanone C2 stereogenic center. The parent-structure drawings and SMILES do not specify an absolute configuration, and no comparative enantiomeric activity is inferred. Parent-structure ADME descriptors are distinguished from the protonated donepezil microspecies used for docking and molecular dynamics.

Molecules were initially drawn in ChemDraw (version 16.0; PerkinElmer, Inc., Waltham, MA, USA), converted to canonical SMILES, and then processed through two web ADME suites. SwissADME [16] furnished essential physicochemical indices and drug-likeness checks based on Lipinski’s rules; pkCSM [17], meanwhile, produced graph-based estimates for human intestinal uptake, CYP450 enzyme metabolism, and renal elimination. The integrated readout formed a multidimensional ADME signature for each candidate, providing model-dependent hypotheses for subsequent experimental assessment.

### 4.2. Prediction of Toxicity Using ProTox-III

Toxicity assessments were carried out in ProTox-III [18]. Canonical SMILES strings, validated in ChemDraw, were uploaded to the platform to obtain predicted acute oral LD_50_, toxicity class, and organ- and pathway-associated alerts. Predicted endpoint labels, probabilities, and applicability indicators were interpreted together. These outputs describe model-based hazards and do not establish measured toxicity, clinical safety, or an experimentally validated mechanism.

### 4.3. PyRx and AutoDock Vina

Ligands were sketched in ChemDraw 22.0, exported as SDF, energy-minimized in PyRx 0.9.9, and converted to PDBQT format. M1, M2, M3, and M4 were modeled with a net charge of 0, whereas donepezil (Dz) was protonated at its tertiary amine, with a net charge of +1. The target protein (PDB ID: 7B2W) was prepared in AutoDockTools 1.5.7 by removing crystallographic waters, adding polar hydrogens, and assigning Gasteiger charges. The original focused docking calculations used a grid centered at x = 2.50, y = 62.46, z = 68.85 Å, with dimensions of x = 8.25, y = 13.16, z = 12.93 Å, and exhaustiveness = 50, applied identically to M1–M4 and Dz. Top-ranked poses were clustered and visually inspected using BIOVIA Discovery Studio 2024 [33]. This focused search volume was used to examine local ligand accommodation within the AChE gorge. Applying identical grid and search settings to M1–M4 and Dz provided a common computational framework for comparing predicted binding poses and interactions within the selected region, consistent with the study’s objective of exploring structure-dependent recognition across the chromone series.

Because 7B2W does not contain a co-crystallized organic inhibitor, same-structure inhibitor redocking was not applicable. Docking validation was therefore benchmarked against the donepezil complex of human AChE (PDB 4EY7). Donepezil was extracted from the crystallographic complex and redocked into 4EY7. The human enzyme was structurally aligned to the fixed 7B2W receptor, and the crystallographic donepezil coordinates were transformed into the 7B2W coordinate frame. Donepezil was subsequently cross-docked into 7B2W and compared with the transferred crystallographic reference pose. The resulting validation outcomes are reported in Section 2.1 and Figure 4.

### 4.4. Molecular Dynamics Simulations

Molecular dynamics simulations were carried out with GROMACS 2023.3 [49]. Protein topologies were generated using gmx pdb2gmx with the AMBER99SB-ILDN force field, which added missing hydrogen atoms and assigned standard protonation states according to the pdb2gmx workflow. Ligands were parameterized using ACPYPE (version 2023.10.27) (Antechamber/AmberTools interface) to generate a GROMACS-compatible topology (.itp) and coordinate (.gro) files, employing GAFF atom types and AM1-BCC partial charges. M1, M2, M3, and M4 were each simulated as a neutral microspecies (net charge = 0), whereas donepezil was simulated as its tertiary-ammonium microspecies (net charge = +1). These fixed protonation states represent the selected forms at pH 7.4; no constant-pH MD protocol or ionization-state switching was applied. Protein–ligand complexes were solvated in a cubic TIP3P water box under three-dimensional periodic boundary conditions (pbc = xyz) and electrically neutralized by adding counter-ions using the Genion tool (ion addition prepared using ions.mdp).

Energy minimization was performed using the steepest descent algorithm (EM.mdp; integrator = steep, nsteps = 50,000, emtol = 1000 kJ·mol^−1^·nm^−1^, emstep = 0.01). Equilibration consisted of a 100 ps NVT phase, followed by a 100 ps NPT phase (NVT.mdp and NPT.mdp), both performed using the leap-frog integrator (integrator = md) with a 2 fs time step (dt = 0.002 ps). Temperature was maintained at 300 K using the V-rescale thermostat (tcoupl = V-rescale, tau_t = 0.1 ps), with separate coupling groups for Protein_LIG and Water_and_ions. Pressure coupling during NPT equilibration was achieved using the Berendsen barostat (pcoupl = Berendsen, isotropic coupling, ref_p_ = 1.0 bar, tau_p_ = 2.0 ps, compressibility = 4.5 × 10^−5^ bar^−1^), followed by an 800 ns production simulation under the Parrinello–Rahman barostat (pcoupl = Parrinello–Rahman, isotropic, ref_p_ = 1.0 bar, tau_p_ = 2.0 ps, compressibility = 4.5 × 10^−5^ bar^−1^).

In all MD stages, bonds involving hydrogen atoms were constrained (constraints = h-bonds) using the LINCS algorithm (constraint_algorithm = lincs, lincs_iter = 1, lincs_order = 4). Nonbonded interactions were treated using the Verlet cutoff scheme (cutoff-scheme = Verlet, nstlist = 20) with a neighbor-list cutoff of rlist = 1.2 nm. Long-range electrostatics were calculated using the Particle Mesh Ewald (PME) method (rcoulomb = 1.2 nm, pme_order = 4, fourierspacing = 0.16 nm), and van der Waals interactions were truncated at 1.2 nm (rvdw = 1.2 nm), with a force-switch modifier applied from 1.0 nm (rvdw-switch = 1.0 nm; vdw-modifier = force-switch). Long-range dispersion correction was not applied (DispCorr = no). Coordinates were saved every 10 ps during production (nstxout-compressed = 5000) for subsequent structural and energetic analyses.

One 800 ns production trajectory was generated for each complex; independent replicate trajectories were not performed. Consequently, the dynamical comparisons describe the individual sampled trajectories, and neither their length nor bounded structural metrics establish reproducibility across independent simulations. PCA/FEL and DCCM analyses are interpreted as exploratory descriptions rather than as replicated evidence of ligand-specific dynamic mechanisms.

### 4.5. MM/PBSA Binding Free Energy Calculations

Binding free energies were estimated exclusively via the molecular mechanics/Poisson–Boltzmann surface area (MM/PBSA) approach using MMPBSA.py from AmberTools 23 in parallel mode. For each protein–ligand complex, 250 snapshots were extracted at uniform 100 ps intervals from the terminal 775–800 ns segment of the 800 ns GROMACS trajectory. This common late-stage window standardized elapsed simulation time across systems; it did not impose identical conformational ensembles or establish equilibration. Specifically, the window follows the late M1 rearrangement and describes its post-transition sample. No additional-window or block-convergence test was performed, so independence of the energetic ordering from the selected 25 ns interval was not established.

For each snapshot, the binding free energy was calculated as the sum of the gas-phase molecular-mechanics terms (ΔE_vdW_ + ΔE_elec_) and the solvation terms (ΔG_PB_ + ΔG_surf_). The Poisson–Boltzmann calculation used an internal dielectric constant of 1.0, an external dielectric constant of 80.0, and an ionic strength of 0.15 M; the nonpolar solvation contribution was estimated from the solvent-accessible surface area. Component energies and ΔG_bind_ are reported as mean ± standard deviation over the 250-snapshot ensemble. Configurational entropy was not evaluated; accordingly, the values are interpreted as comparative end-state estimates rather than as absolute experimental binding free energies [43].

### 4.6. Density Functional Theory Calculations

All quantum-chemical calculations were performed with ORCA 5.0.4 under an implicit aqueous environment using the CPCM(water) solvation model. The four chromone derivatives, M1–M4, were treated as neutral closed-shell singlets (q = 0; multiplicity = 1). Ground-state geometries were optimized at the B3LYP-D3BJ/def2-SVP level using the ORCA keyword line ! B3LYP D3BJ def2-SVP Opt TightSCF CPCM(water). Harmonic frequency calculations were then carried out to confirm that the optimized structures correspond to true minima (no imaginary frequencies). HOMO and LUMO energies were extracted from the converged wavefunctions and used to compute global electronic/reactivity descriptors.

For real-space analyses, ORCA wavefunctions were exported in Molden format and processed with Multiwfn 3.8. ELF maps and MEP grids/surfaces were generated directly in Multiwfn using identical grid settings across all compounds. HOMO/LUMO isosurfaces and MEP surfaces were visualized in Avogadro 1.2.0 with consistent rendering parameters to enable direct cross-comparison. NCI plots derived from Multiwfn outputs were finalized at high resolution using GIMP 3.0.6-1 to ensure publication-quality figures.

## 5. Conclusions

This study evaluated four designed chromone derivatives as computational candidates for AChE-directed investigation. The absence of a co-crystallized organic inhibitor in 7B2W was addressed by benchmarking donepezil redocking in human 4EY7 and cross-docking against the crystallographic reference transferred to 7B2W. These tests support reference-pose recovery, but do not establish human inhibition by M1–M4, identical species-specific binding, or full CAS–PAS coverage by the original focused docking grid.

Docking and long-timescale molecular dynamics demonstrated that ligand recognition cannot be adequately described by a single static pose. The chromone derivatives displayed distinct patterns of conformational adaptation and interaction persistence within the AChE gorge. Ensemble-based MM/PBSA analysis gave M3 the most favorable mean chromone estimate, within 775–800 ns, whereas M4 earned the most favorable chromone docking score. PCA/FEL and DCCM further revealed ligand-dependent conformational states and collective-motion networks, describing sampled differences rather than replicated or causal effects of ligand identity.

DFT calculations provided a complementary description of the electronic properties of the compounds, while ADME and toxicity predictions demonstrated that lead selection should integrate binding behavior, dynamic stability, pharmacokinetic properties, and potential safety liabilities rather than rely on a single computational criterion.

Overall, M3 and M4 are computationally prioritized candidates, not experimentally established AChE inhibitors. Their complementary prioritization rests on different criteria, and the single-trajectory design and single-terminal MM/PBSA window preclude claims of replicated dynamics or a temporally robust energetic ranking. No experimental AChE inhibition, kinetic mechanism, nor effect on Aβ assembly have been established. Enzyme-inhibition assays, followed by selectivity, permeability, and safety measurements, are required before claims of biological activity or therapeutic relevance can be validated.

## Figures and Tables

**Table 1:** Physicochemical descriptors and drug-likeness indices (SwissADME) for candidate flavones (M1–M4) compared with reference inhibitor donepezil.

Molecules	1	2	3	4	Dz
Molecular weight (g/mol)	282.29	338.4	280.32	310.34	379.49
H-bond acceptors	4	4	3	4	4
H-bond donors	0	0	0	0	0
Rotatable bonds	3	3	2	4	6
TPSA (Å^2^)	48.67	48.67	39.44	48.67	38.77
CLogP	3.15	4.36	3.81	3.85	4
Lipinski violations	0	0	0	0	0
Veber violations	0	0	0	0	0
Bioavailability Score	0.55	0.55	0.55	0.55	0.55

**Table 2:** pkCSM metabolic profile of chromone derivatives (M1–M4) versus that for donepezil (Dz): CYP450 substrate/inhibitor status, renal OCT2 engagement and total clearance (log mL min^−1^ kg^−1^).

Molecules	M1	M2	M3	M4	Dz
CYP2D6 substrate	No	No	No	No	No
CYP3A4 substrate	Yes	Yes	Yes	Yes	Yes
CYP2D6 inhibitor	No	No	No	No	Yes
CYP3A4 inhibitor	No	No	No	No	Yes
Renal OCT2 substrate	Yes	Yes	Yes	Yes	Yes
Total Clearance (log mL/min/kg)	0.676	0.657	0.614	0.447	0.998

**Table 3:** Complete ProTox-III target-level toxicity predictions for chromone derivatives M1–M4 and donepezil (Dz). Each cell reports the predicted status and probability (%); A, active; I, inactive.

ProTox-III Target/Endpoint	M1	M2	M3	M4	Dz
Organ toxicity					
Hepatotoxicity	I (73%)	I (72%)	I (73%)	I (66%)	I (98%)
Neurotoxicity	I (67%)	I (67%)	I (50%)	I (73%)	A (90%)
Nephrotoxicity	A (51%)	A (52%)	A (51%)	A (55%)	I (67%)
Respiratory toxicity	A (60%)	A (57%)	A (58%)	A (60%)	A (96%)
Cardiotoxicity	I (64%)	I (73%)	I (72%)	I (65%)	I (67%)
Toxicity end points					
Carcinogenicity	A (56%)	I (52%)	A (50%)	I (55%)	A (50%)
Immunotoxicity	I (90%)	A (57%)	I (97%)	I (82%)	A (95%)
Mutagenicity	I (51%)	I (71%)	I (70%)	I (67%)	I (53%)
Cytotoxicity	I (57%)	I (75%)	I (52%)	I (92%)	A (63%)
BBB	A (61%)	A (67%)	A (68%)	A (69%)	A (99%)
Ecotoxicity	A (71%)	A (77%)	A (79%)	A (67%)	A (56%)
Clinical toxicity	I (56%)	I (60%)	I (62%)	I (66%)	A (56%)
Nutritional toxicity	I (53%)	A (60%)	A (59%)	A (57%)	I (53%)
Tox21 Nuclear-receptor signaling pathways					
Aryl hydrocarbon Receptor (AhR)	A (70%)	A (89%)	A (88%)	A (76%)	I (81%)
Androgen Receptor (AR)	I (98%)	I (98%)	I (99%)	I (97%)	I (96%)
Androgen Receptor Ligand Binding Domain (AR-LBD)	I (99%)	I (99%)	I (99%)	I (98%)	I (99%)
Aromatase	I (67%)	I (64%)	I (62%)	I (68%)	I (89%)
Estrogen Receptor Alpha (ER)	A (66%)	A (66%)	A (67%)	I (55%)	A (58%)
Estrogen Receptor Ligand Binding Domain (ER-LBD)	I (82%)	I (72%)	I (82%)	I (84%)	I (98%)
Peroxisome Proliferator Activated Receptor Gamma (PPAR-Gamma)	I (88%)	I (98%)	I (98%)	I (94%)	I (99%)
Tox21 Stress-response pathways					
Nuclear factor (erythroid-derived 2)-like 2/antioxidant responsive element (nrf2/ARE)	I (96%)	I (90%)	I (93%)	I (89%)	I (98%)
Heat Shock Factor Response Element (HSE)	I (96%)	I (90%)	I (93%)	I (89%)	I (98%)
Mitochondrial Membrane Potential (MMP)	I (57%)	A (69%)	A (57%)	A (52%)	I (88%)
Phosphoprotein (Tumor Suppressor) p53	I (90%)	I (81%)	I (83%)	I (82%)	I (94%)
ATPase family AAA domain-containing protein 5 (ATAD5)	A (68%)	A (82%)	A (85%)	I (67%)	I (99%)
Molecular Initiating Events					
Thyroid hormone receptor alpha (THRα)	I (87%)	I (82%)	I (87%)	I (79%)	I (65%)
Thyroid hormone receptor beta (THRβ)	I (74%)	I (86%)	I (83%)	I (84%)	I (70%)
Transthyretin (TTR)	A (84%)	A (86%)	A (89%)	A (81%)	I (91%)
Ryanodine receptor (RYR)	I (95%)	I (95%)	I (95%)	I (94%)	I (78%)
GABA receptor (GABAR)	A (52%)	A (62%)	A (63%)	A (60%)	I (66%)
Glutamate N-methyl-D-aspartate receptor (NMDAR)	I (99%)	I (99%)	I (99%)	I (99%)	I (81%)
alpha-amino-3-hydroxy-5-methyl-4-isoxazolepropionate receptor (AMPAR)	I (100%)	I (100%)	I (100%)	I (100%)	I (100%)
Kainate receptor (KAR)	I (100%)	I (100%)	I (100%)	I (100%)	I (100%)
Acetylcholinesterase (AChE)	A (50%)	I (56%)	I (51%)	I (58%)	A (98%)
Constitutive androstane receptor (CAR)	I (93%)	I (99%)	I (99%)	I (99%)	I (100%)
Pregnane X receptor (PXR)	I (57%)	I (56%)	I (59%)	I (62%)	I (77%)
NADH-quinone oxidoreductase (NADHOX)	I (87%)	I (85%)	I (86%)	I (76%)	I (74%)
Voltage gated sodium channel (VGSC)	I (82%)	I (89%)	I (89%)	I (89%)	I (85%)
Na+/I- symporter (NIS)	I (92%)	I (95%)	I (95%)	I (95%)	I (95%)
Metabolism					
Cytochrome CYP1A2	A (97%)	A (84%)	A (84%)	A (73%)	I (51%)
Cytochrome CYP2C19	A (86%)	A (66%)	A (69%)	A (62%)	I (74%)
Cytochrome CYP2C9	A (87%)	A (51%)	A (60%)	A (62%)	I (61%)
Cytochrome CYP2D6	I (75%)	I (74%)	I (74%)	I (72%)	A (86%)
Cytochrome CYP3A4	A (90%)	I (63%)	I (50%)	I (56%)	I (53%)
Cytochrome CYP2E1	I (96%)	I (99%)	I (99%)	I (99%)	I (99%)

**Table 4:** Docking scores, reported polar contacts and intermolecular distances for chromone derivatives (M1–M4) compared with the results for donepezil bound to acetylcholinesterase.

Molecules	Docking Score (kcal/mol)	Reported Polar Contacts	Distance (Å)
M1	−9	Tyr130, Glu199, Ser200	3.52; 3.49; 2.82
M2	−9.5	_	_
M3	−9.2	_	_
M4	−9.6	Gly117, Gly118, Tyr121, Glu199	3.77; 2.49; 2.35; 3.72
Dz	−9.3	Tyr121, Arg289	2.57; 3.38

**Table 5:** MM/PBSA binding free-energy components calculated from 250 snapshots extracted over 775–800 ns (mean ± SD; kcal/mol).

Ligand	ΔE_vdW_	ΔE_elec_	ΔG_PB_	ΔG_surf_	ΔG_gas_	ΔG_solv_	ΔG_bind_
M1	−32.47 ± 4.54	−5.69 ± 3.62	22.58 ± 6.22	−3.27 ± 0.36	−38.16 ± 6.57	19.31 ± 5.95	−18.85 ± 2.87
M2	−37.94 ± 2.02	−6.93 ± 2.60	28.61 ± 3.46	−3.88 ± 0.13	−44.87 ± 3.41	24.73 ± 3.48	−20.14 ± 2.79
M3	−38.31 ± 1.84	−14.79 ± 3.33	32.97 ± 3.13	−3.77 ± 0.09	−53.10 ± 3.36	29.20 ± 3.10	−23.90 ± 2.31
M4	−38.88 ± 2.01	−8.73 ± 2.52	35.76 ± 5.20	−4.09 ± 0.10	−47.62 ± 3.40	31.66 ± 5.16	−15.95 ± 3.86
Dz	−45.65 ± 2.71	−264.24 ± 7.27	273.53 ± 7.22	−4.64 ± 0.17	−309.89 ± 7.56	268.89 ± 7.19	−41.00 ± 2.75

**Table 6:** DFT-derived global reactivity descriptors of M1–M4: HOMO/LUMO energies, ΔE(HOMO–LUMO), hardness (η), softness (S), chemical potential (μ), and electrophilicity index (ω).

Descriptor	M1 (eV)	M2 (eV)	M3 (eV)	M4 (eV)
HOMO energy	−6.031	−5.873	−6.017	−6.221
LUMO energy	−1.897	−1.768	−1.817	−1.895
Energy gap (HOMO–LUMO)	+4.134	+4.105	+4.199	+4.326
Hardness (η)	+2.067	+2.053	+2.099	+2.163
Softness (S, eV^−1^)	+0.484	+0.487	+0.476	+0.462
Chemical potential (μ)	−3.964	−3.821	−3.917	−4.058
Electrophilicity index (ω)	+3.801	+3.556	+3.654	+3.807

Equations used: Egap = E_LUMO_ − E_HOMO_; η (hardness) = Egap/2; S (softness) = 1/η [units: eV^−1^]; μ (chemical potential) = (E_HOMO_ + E_LUMO_)/2; ω (electrophilicity) = μ^2^/(2η).

## Data Availability

All data and computational files supporting the findings of this study are openly available in the public GitHub repository “Chromone-Scaffolds” at: https://github.com/khibech/Chromone-Scaffolds- (accessed on 25 August 2026). The repository includes the ORCA input (.inp) and Molden (.molden) files for the investigated compounds, as well as the main GROMACS molecular dynamics files, including topology and coordinate files (.itp, .top, and .gro) and the corresponding analysis output files (.xvg). In addition, the repository contains the relevant MM/PBSA input (.inp) and output/data (.dat) files used for the binding free energy calculations. These files are freely accessible and are provided to ensure transparency, reproducibility, and independent verification of the computational procedures and results reported in this study.

## Abbreviations

AD	Alzheimer’s Disease
AChE	Acetylcholinesterase
CNS	Central Nervous System
ADMET	Absorption, Distribution, Metabolism, Excretion, and Toxicity
MD	Molecular Dynamics
MM/PBSA	Molecular Mechanics/Poisson–Boltzmann Surface Area
RMSD	Root Mean Square Deviation
RMSF	Root Mean Square Fluctuation
Rg	Radius of Gyration
TPSA	Topological Polar Surface Area
H-bonds	Hydrogen Bonds
VDss	Steady-State Volume of Distribution
logBB	Logarithmic Blood–Brain Barrier Penetration
logPS	Logarithmic CNS Permeability
P-gp	P-glycoprotein
SwissADME	Swiss Absorption, Distribution, Metabolism, and Excretion
pkCSM	Pharmacokinetic Chemistry Space Modeling
ProTox-III	Prediction of Toxicity
AutoDock Vina	Molecular Docking Software
GROMACS	Groningen Machine for Chemical Simulations
ADT	AutoDock Tools
SAS	Solvent Accessible Surface
